# Supplementary material for: DAGFormer: A graph-based domain adaptation approach for single-cell cancer drug response prediction
Source: PLoS Comput Biol. 2025 Dec 19;21(12):e1013832. doi: 10.1371/journal.pcbi.1013832 (PMC12795466; doi:10.1371/journal.pcbi.1013832)
Supplement: Attachment — Submitted filename: Response to Reviewers.pdf [file pcbi.1013832.s002.pdf]

Authors' Response to Reviewers' Comments

**Paper title:**	DAGFormer: A graph-based domain adaptation approach for single-cell cancer drug response prediction
**Authors:**	Fen Yan, ZhiHua Du, Yu-An Huang

We would like to express our sincere gratitude to the editor and the reviewers for their careful review of the previous version of our manuscript. We are pleased to inform you that we have completed the revision of the manuscript according to your comments and suggestions.

Reviewer 1

The manuscript by Yan, Du, and Huang presents DAGFormer, an graph-based domain adaptation framework for integrating bulk RNA-seq and scRNA-seq data to predict cancer drug responses at the single-cell level. The prediction of single-cell drug response is crucial for advancing personalized cancer therapy, and the challenges of integrating heterogeneous data sources (bulk vs. single-cell) are well-recognized. The manuscript is well-written and clearly articulates this significance. I recommend acceptance after revisions, as outlined below.

Response: We thank the reviewer for the time and effort to review our manuscript giving a number of suggestions to improve the paper.

**Comment 1:**
1. In Figure 1, the bulk RNA-seq dataset (Table 1) is used as the source domain and scRNA-seq datasets (Table 2) as the target domain. Please clarify whether the scRNA-seq data are used exclusively for evaluation or also involved during training.

Response: We thank you for this insightful question, which addresses a fundamental aspect of our Graph-based Domain Adaptation (GDA) framework.

The single-cell RNA-seq (scRNA-seq) data from Table 2 serves two distinct but interconnected roles within the DAGFormer framework, which is the core principle of Unsupervised Domain Adaptation (UDA):

1. **Involvement in Training (Target Domain Data)**: The scRNA-seq expression profiles (X_t) are actively involved in the training process. Crucially, the drug response labels (Y_t) for this target domain are not used during training. The scRNA-seq data are incorporated exclusively as unlabeled target-domain samples. Specifically, the scRNA-seq data is utilized to:

- Align Feature Distributions: We employ the Graph Domain Adaptation (GDA) component, which uses the scRNA-seq feature representations to minimize the distribution shift between the source (Bulk RNA-seq) and target (scRNA-seq) domains. This alignment allows knowledge transfer from the labeled source domain.

- Promote Smoothness and Confidence: We use the Cross-entropy Minimization Loss ($\mathcal{L}_{\text{ent}}$)on the scRNA-seq data to encourage high-confidence predictions on the target domain features, leveraging the graph structure(G_t) .

2. **Exclusive Use for Evaluation:** The corresponding, unseen drug response labels (Y_t) for the scRNA-seq data are strictly held out and are only used for the final evaluation (to calculate metrics like AUC, AUPR). This ensures an unbiased assessment of the model's generalization capability to new, unseen single-cell data.

In summary, the scRNA-seq expression features are part of the unsupervised training to facilitate domain transfer, while the corresponding labels are exclusively reserved for rigorous evaluation. In addition, to formally clarify this distinction and ensure maximum reader comprehension, we have added a dedicated section in the revised manuscript: Section 2.1 Problem Definition.

"This paper addresses the binary classification task of predicting single-cell cancer drug response. Our primary goal is to leverage rich label information from large-scale cell line data (Bulk RNA-seq) to accurately forecast drug sensitivity at the single-cell level......Therefore, effectively transferring label knowledge and bridging this graph-level domain gap without relying on target domain labels (Y_t) is the central challenge of this work." ***(Page 4, Line 103-133)***

**Comment 2:**
2. The overall loss function includes several weighting coefficients. Please provide details on how these hyperparameters were selected (e.g., grid search, prior work, or manual tuning). In addition, specify the computational environment (e.g., GPU model, hardware configuration) to support reproducibility.

Response: We sincerely thank you for the valuable comment. Our total loss function is formulated as $\mathcal{L} = \mathcal{L}_{\text{cls}} + \lambda_d \mathcal{L}_{\text{dom}} + \lambda_e \mathcal{L}_{\text{ent}} + \lambda_f \mathcal{L}_{\text{diff}} + \lambda_r \mathcal{L}_{\text{rec}}$.

1. Hyperparameter selection:

The weighting coefficients were determined by manual grid search guided by values commonly used in adversarial and domain adaptation frameworks.

Specifically, we evaluated a small set of candidate combinations based on prior work and empirical performance stability across different datasets. After empirical evaluation, we used the following configuration for all experiments to ensure consistency:

$$\lambda_d = 1.0,\ \lambda_r = 0.3,\ \lambda_f = 1 \times 10^{-4}.$$

The entropy term coefficient was linearly scheduled according to training progress: $\lambda_e(p) = 0.01 \times p$, (implemented as (implemented as loss_entropy * (epoch/n_epoch * 0.01)), while the gradient reversal strength was scheduled as rate = min((epoch+1)/n_epoch, 0.05).

2. Computational environment and reproducibility.

All experiments were conducted on a single workstation configured as follows:

- Hardware: NVIDIA GeForce RTX 3090 GPU (24 GB VRAM), Intel Xeon 16-core CPU, 128 GB RAM.

- Operating system: Ubuntu 22.04 LTS.
- Software stack: Python 3.10, PyTorch 2.4.1 (CUDA 12.1, cuDNN 8.9), DGL 2.4.0+cu121, torch-geometric 2.6.1, torch-scatter 2.1.2+pt24cu121, torch-sparse 0.6.18+pt24cu121, scikit-learn ⩾1.3, NumPy ⩾1.24.
- Training details: optimizer Adam (lr = 1e-2, weight_decay = 5e-4), batch size 256, 400 epochs, early stopping disabled unless specified.

To ensure the full reproducibility of our results, the code, datasets, and training scripts for the DAGFormer framework are publicly available at https://github.com/yanfen-git/DAGFormer. We have thoroughly updated the README.md file in this repository to specify the detailed computational environment, including the exact hardware setup and software dependencies. This update facilitates the exact replication of all reported experiments by providing installation requirements and example command-line usage.

"The stability and reliability of the proposed Graph Domain Adaptation (GDA) framework were rigorously established through a systematic sensitivity analysis focused on the core weighting coefficients......This dynamic strategy ensures that the entropy constraint gradually intensifies only as the feature alignment and classification knowledge mature, guaranteeing a more robust and stable UDA training progression" ***(Page 24, Line 777-812)***

**Comment 3:**
3. Figure 5 visualization: The color scheme of Figure 5 could be improved for consistency and readability. A unified and aesthetically optimized palette would enhance clarity.

Response: Thank you for your valuable feedback regarding the clarity and presentation of our figures. We agree that high-quality visuals are essential for effective scientific communication.

We have comprehensively improved the visualization of Figure 5 to enhance consistency and readability:

1. Unified Color Palette and Contrast: We have adopted a single, aesthetically optimized color palette across all panels. Specifically, in Panel B (Comparison with alternative graph encoders), we have ensured a palette with greater contrast to clearly distinguish each model. The Graph Transformer (Ours) is highlighted using a distinct color to maximize visual attention.

2. Removal of Visual Clutter: We have removed unnecessary gray horizontal grid lines from the figure, which significantly reduces visual clutter and improves focus on the data distribution.

3. Consistency in Labeling: We have ensured that the colors representing performance metrics (ACC, AUC, AUPR) in Panel A (Training Size) are distinct and consistent across all drug subplots.

These comprehensive improvements significantly enhance the aesthetic quality, consistency, and overall interpretability of Figure 7 in the revised manuscript.

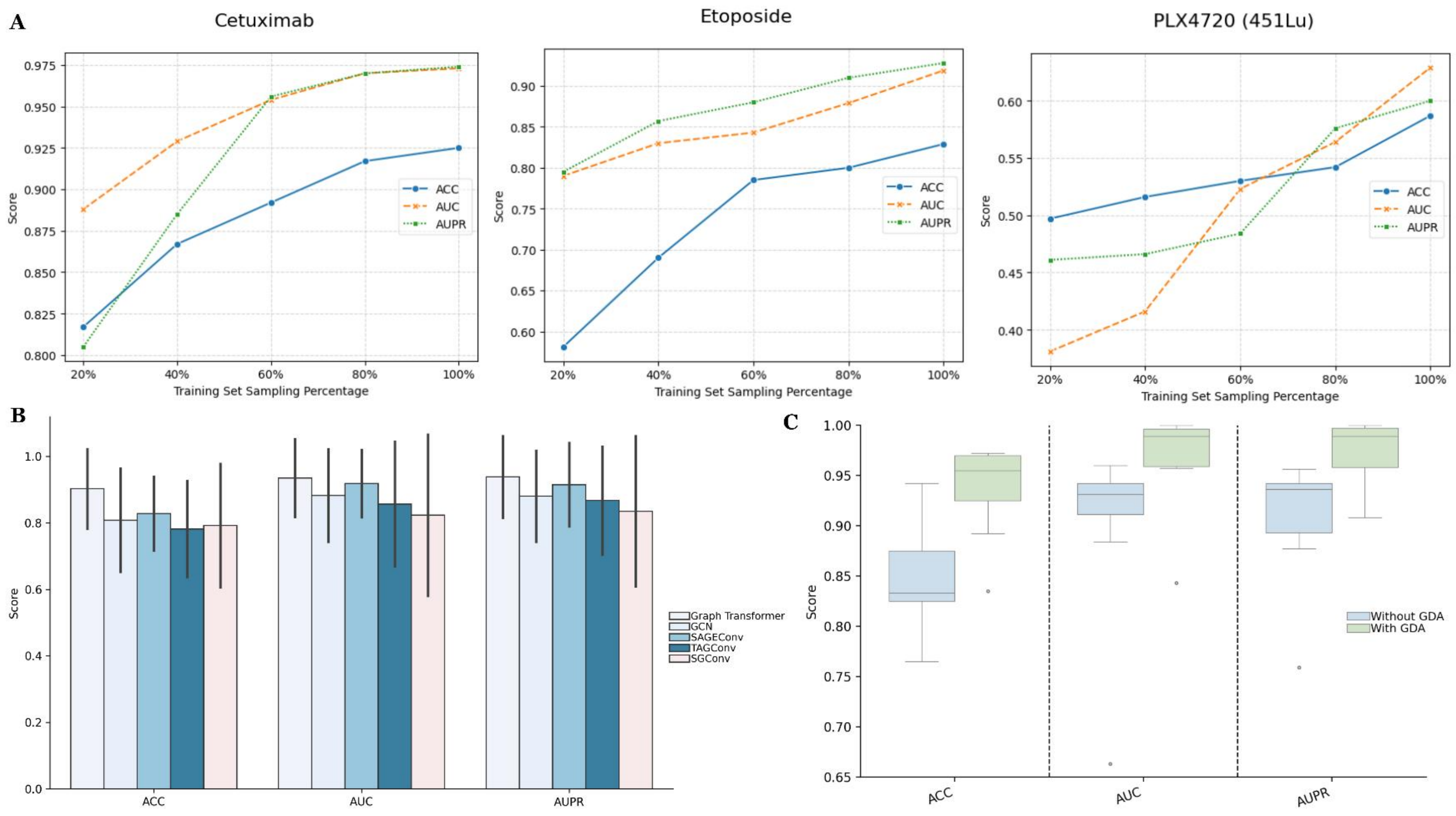

Fig 7. Comprehensive evaluation of DAGFormer. (A) Performance under varying training set sizes; (B) Comparison with alternative graph encoders; (C) Ablation analysis of the Graph-based Domain Adaptation (GDA) mechanism.

**Comment 4:**
4. Minor language issue: On page 3, line 76, “Bulk RNA-seq” is capitalized incorrectly. For consistency, please revise to “bulk RNA-seq” (lowercase, unless at the beginning of a sentence).

Response: We appreciate you noting this minor but important language inconsistency. We confirm that we will revise the capitalization on page 3, line 76 (and throughout the entire manuscript where applicable) from the incorrect "Bulk RNA-seq" to the proper style: "bulk RNA-seq" (lowercase, unless beginning a sentence). This ensures consistency and adherence to standard scientific nomenclature.

Reviewer 2

This manuscript by Yan et al. presents DAGFormer, a novel deep learning framework for predicting single-cell drug responses by integrating bulk and single-cell RNA-seq data. The authors address key limitations of 2 previous methods by 1) incorporating cellular relationship graphs as constraints when encoding features of each cell/cell-line and 2) employing a dual-domain architecture to preserve both domain-shared and domain-private features, specifically accomplished via a combination of an adversarial training scheme on shared features and enforcing orthogonality between shared and private features.

Through comprehensive experiments on 9 different drugs, the authors demonstrate that DAGFormer significantly outperforms a suite of classical and state-of-the-art baseline models. The authors further present ablation studies to provide adequate support and reasoning for their model setup and choice. This work presents an improved approach to transferring characteristics learnt from bulk RNA-seq data to scRNA-seq.The results are compelling, though are several points of concern as listed below.

Response: We would like to thank the Reviewer for taking the time and effort necessary to review the manuscript.

Comment 1:

1. Unclear training and testing scheme. it is unclear how the training data and test data for each drug was set up. From the results and data descriptions, it seems that the scRNA-seq data was only used for testing and evaluations, however the model explicitly calculates losses for scRNA-seq derived features such as the graph reconstruction loss and adversarial loss between bulk and single cell embeddings. The authors should provide a detailed description of how training and testing data was set up for a drug/experiment (assuming it is the same for the other drugs).

Response: We sincerely thank you for raising this crucial question. We understand that the distinction between the data used for unsupervised training losses and the data used for final performance evaluation requires explicit clarification. The overall training and testing scheme follows a standard Unsupervised Domain Adaptation (UDA) paradigm, where we transfer knowledge from a labeled source domain (Bulk RNA-seq) to an unlabeled target domain (scRNA-seq) for training, followed by a held-out evaluation.

1. Detailed Training and Testing Scheme Setup

For every drug experiment (e.g., Gefitinib, Etoposide, etc.), the data were partitioned as follows:

Table R2-1

Data Type	Composition	Usage During Training	Usage During Evaluation
Bulk RNA-seq(Source domain)	Gene expression (X_s) and Drug Response Labels (Y_s)	Supervised Training: Optimizes the Classification Loss ($\mathcal{L}_{cls}$). Joint Training: Contributes features to $\mathcal{L}_{dom}$, $\mathcal{L}_{diff}$, $\mathcal{L}_{rec}^{1}$.	Not used.
scRNA-seq(Target Domain)	Gene expression (X_t) [Unlabeled]	Unsupervised Training: Optimizes the Entropy Loss ($\mathcal{L}_{ent}$). Joint Training: Contributes features to $\mathcal{L}_{dom}$, $\mathcal{L}_{diff}$, $\mathcal{L}_{rec}^{2}$.	Exclusive Testing: The held-out labels (Y_t) are used only to compute final metrics (ACC, AUC, AUPR) and assess model generalization.

In addition, to formally clarify this distinction and ensure maximum reader comprehension, we have added a dedicated section in the revised manuscript: Section 2.1 Problem Definition.

2. Clarifying Unsupervised and Joint Loss Calculation

The explicit calculation of losses using scRNA-seq-derived features (X_t) is the core mechanism of UDA. This process enables feature alignment and structural preservation without needing target labels (Y_t).

- Domain Adaptation Loss ($\mathcal{L}_{dom}$): This adversarial loss is computed using the shared features from both source domain and target domain. It is the

mechanism that drives feature alignment, making the Bulk and scRNA-seq embeddings indistinguishable to the domain discriminator. This aligns distributions without requiring Y_t.

- Difference Loss ($\mathcal{L}_{\text{diff}}$): This loss is calculated on features from both source domain and target domain. Its purpose is to enforce orthogonality between the shared features (H^{sh}) and the private features (H^{pr}) in each domain, ensuring domain-specific biological information is preserved while invariant features are disentangled.

- Graph Reconstruction Loss ($\mathcal{L}^2_{\text{rec}}$): This loss ensures the encoders learn representations that accurately reconstruct the input cellular neighbor graph G_t. It uses the graph G_t and the reconstructed graph $\widetilde{G}_t$, not the drug response label Y_t.

- Entropy Loss ($\mathcal{L}_{\text{ent}}$): This loss minimizes the uncertainty (entropy) of the predictions on the scRNA-seq data , pushing the model toward high-confidence outputs. It operates only on the unlabeled predictions.

"This paper addresses the binary classification task of predicting single-cell cancer drug response. Our primary goal is to leverage rich label information from large-scale cell line data (Bulk RNA-seq) to accurately forecast drug sensitivity at the single-cell level......Therefore, effectively transferring label knowledge and bridging this graph-level domain gap without relying on target domain labels (Y_t) is the central challenge of this work." ***(Page 4, Line 103-133)***

"The adversarial training process utilizes a domain adversarial loss ......ensuring feature invariance" ***(Page 13, Line 453-458)***

**Comment 2:**
2. As a follow-up to the previous point, if all single cells were utilized for training (similar to scDEAL), it would be interesting to see the results of a portion of single cells that are left out of training entirely (no contribution to model losses) and only used for evaluation (especially for drugs like Etoposide that have more single cells).

Response: Thank you for your valuable feedback. We greatly appreciate this insightful follow-up suggestion. You raises a critical point concerning the robustness of our Unsupervised Domain Adaptation (UDA) model when faced with large target domain datasets, particularly the comparison between using all single cells for

unsupervised training versus reserving a truly independent, unseen subset for final evaluation.

To rigorously address this concern and demonstrate the generalization ability of DAGFormer, we performed a new ablation experiment specifically for the Etoposide dataset, as it contains a substantial number of single cells.

We modified our training scheme for this experiment. First, we partitioned the original Etoposide scRNA-seq data ($\mathrm{D_T}$) into a Training Target Set ($\mathrm{D_T^{train}}$) and an Independent Hold-out Set ($\mathrm{D_T^{test}}$), using an 80%/20% split (Modified Setup). Second, during the Training Phase, only the $\mathrm{D_T^{train}}$ set was used for unsupervised losses ($\mathcal{L}_{\mathrm{ent}}$, $\mathcal{L}_{\mathrm{dom}}$, $\mathcal{L}_{\mathrm{diff}}$, $\mathcal{L}_{\mathrm{rec}}^2$), ensuring the $\mathrm{D_T^{test}}$ data was entirely excluded from the training process (no contribution to any model losses). Third, in the Evaluation Phase, we compared the model's performance on the new $\mathrm{D_T^{train}}$ set against the performance obtained using the original UDA scheme (where all $\mathrm{D_T}$ was used for unsupervised training and evaluation). The results is presented below:

Table R2-2

Evaluation Metric	Original UDA Scheme (All D_T used in UDA Training)	New Ablation Scheme (20% D_T Held Out)
ACC	0.835	0.728
AUC	0.843	0.803
AUPR	0.908	0.855

The results confirm the successful generalization capability of our GDA mechanism while also highlighting the vital role of the Entropy Loss ($\mathcal{L}_{\mathrm{ent}}$) in optimizing the decision boundary. First, the most reliable metrics for a classification model, AUC and AUPR, show relatively small declines of 0.040 and 0.053, respectively. Crucially, the model maintains a strong absolute performance, with AUC=0.803 and AUPR=0.855 on the entirely unseen data. This outcome confirms the basic effectiveness of our method: the knowledge transfer is successful, and the feature space is highly generalized. Specifically, this demonstrates that even when 20% of the target domain samples are completely unseen during training, the model—by utilizing the GDA mechanism and shared encoder—learns domain-invariant features that retain powerful discriminative ability and reliability. Second, the more pronounced drop in Accuracy (ACC) of 0.107 is primarily attributable to the reduction in the contribution of the $\mathcal{L}_{\mathrm{ent}}$ during training. In the original scheme, $\mathcal{L}_{\mathrm{ent}}$ utilized all target domain data to aggressively sharpen the decision boundary, which led to high-confidence predictions and subsequently boosted the overall ACC score. When 20% of the data is excluded from the training set, the model loses the benefit of this boundary sharpening on the held-out samples, resulting in a noticeable decrease in the final ACC score. This drop therefore reflects the loss of the high-accuracy

gain provided by the density maximization function ($\mathcal{L}_{\text{ent}}$) itself, rather than indicating a fundamental failure of the feature alignment mechanism.

To formally address this critical distinction and demonstrate the robustness of DAGFormer, we have incorporated this detailed analysis and the results of the ablation experiment into a new dedicated subsection in the revised manuscript: Section 3.8, titled "Performance on Independent Hold-out Target Data."

"To rigorously address the concern regarding the generalization capacity of our Unsupervised Domain Adaptation (UDA) framework, particularly when utilizing the entire target dataset for unsupervised training, we conducted a critical ablation experiment to evaluate DAGFormer's performance on a completely independent, unseen subset of single-cell data......provides a beneficial, data-dependent enhancement to prediction confidence." ***(Page 25, Line 813-848)***

Comment 3:

3. It is also unclear whether the authors trained with the mini-batches or the entire data all at once. If mini-batches were used, it would be interesting to see the effect of batch-size on results since smaller batch-sizes may not contain enough cellular relationships to construct meaningful embeddings.

Response: Thank you for raising this important point regarding our training methodology. We appreciate the opportunity to clarify this detail. We confirm that for all reported results, the model was trained using the entire dataset in a single batch (full-batch training), not mini-batches. This approach was chosen and validated as effective due to several factors:

First, the overall size of the aligned gene set across the number of samples (cells/cell lines) in our specific datasets (Tables 1 and 2) allows the entire graph and feature matrices to fit into memory without significant computational overhead. Second, for graph-based learning (Graph Transformer), a key concern with mini-batches is the potential disruption of cellular neighborhood relationships during sampling. By using the full-batch approach, we inherently ensure that the entire cellular neighbor graph (G_s and G_t) is visible and processed simultaneously in every epoch. This guarantees the maximal integrity of global cellular relationships for feature extraction. Third, Full-batch training provides a more accurate and stable estimation of the true gradient compared to mini-batches, which is highly beneficial for stabilizing the complex, non-convex optimization landscape of the Adversarial Domain Adaptation ($\mathcal{L}_{\text{dom}}$) and Disentanglement ($\mathcal{L}_{\text{diff}}$) components.

Your concern that "smaller batch-sizes may not contain enough cellular relationships" is precisely why we chose the full-batch strategy. Our approach eliminates this risk entirely by ensuring 100% of the cellular relationships are seen during every gradient calculation, which maximizes the quality of the learned graph embeddings.

To formally document this methodology in the manuscript, we have added a clarifying statement to the first paragraph of Section 2.6.1 Private encoders for bulk and single-cell data. The added sentence reads: "For all subsequent analyses and training reported, we utilized a full-batch approach to ensure the entire cellular neighbor graph and feature matrices fit into memory and were processed simultaneously in every epoch." ***(Page 10, Line 321-323)***

**Comment 4:**
4. The process of graph construction is still unclear. For instance, is it correct to assume that graph construction for the bulk data was performed with bulk RNA-seq of all cell lines prior to any training and not on-the-fly within each batch, the descriptions are vague.

Response: Thank you for seeking this crucial clarification on the graph construction process. We understand that this step is foundational to our graph-based approach. We confirm that your assumption is entirely correct.

The cellular neighbor graphs are constructed statically (prior to any training) for both the source (Bulk RNA-seq) and target (scRNA-seq) domains, and not on-the-fly within batches. The detailed process is presented below:

1. Bulk Data Graph (G_s): The graph construction (using the K-Nearest Neighbors (KNN) method with K=15, as determined by empirical evaluation) is performed once on the entire population of bulk RNA-seq cell lines (X_s).

2. scRNA-seq Data Graph (G_t): Similarly, the graph is constructed once on the entire population of single cells (X_t) for each specific drug dataset.

3. Training Implementation: Since we use full-batch training (as previously clarified), the entire pre-computed graph (G_s and G_t) is loaded into the GPU memory and utilized by the Graph Transformer in every single training iteration. This strategy ensures that the maximal integrity of cellular relationships is maintained for feature extraction and is never compromised by mini-batch sampling.

To ensure maximal clarity on this fundamental methodology, we have added a concluding statement to Section 2.5 Exploring cellular neighbor graphs construction in the revised manuscript: "For implementation, the cellular neighbor graphs (G_s and G_t) for both the bulk and single-cell populations are constructed statically (i.e., once prior to any training iteration) and are subsequently utilized in a full-batch manner throughout the entire optimization process to preserve the maximal integrity

of cellular relationships." ***(Page 9, Line 286-290)***

**Comment 5:**
5. Unclear description of oversampling and under-sampling (line 127 -line 133), the authors mention both oversampling minority classes to 50% of majority class and under-sampling majority class to 50% of minority class, which does not make sense. If both sampling techniques were used, the authors could include supplementary figures to show the differences in results.

Response: Thank you for pointing out the ambiguity in lines 127–133 regarding our data balancing strategy. We apologize for the unclear wording, which led to a misunderstanding of the target proportions. We confirm that we used a sophisticated data balancing technique — a sequential pipeline combining SMOTE oversampling and Random UnderSampler.

The confusing statement stemmed from a misinterpretation of the target ratios:

1. Original Text Ambiguity: The statement that "oversampling to 50% of the majority class" and "undersampling to 50% of the minority class" was poorly phrased and mathematically contradictory as written.
2. Actual Goal: Our goal was to sequentially adjust the class proportions to reach an intermediate ratio of 50% before the final balancing step, aiming for a more gradual and robust equilibrium between the sensitive (minority) and resistant (majority) cell lines.

The process for achieving balance in the training set works sequentially:

1. Oversampling (SMOTE): The minority class (sensitive cell lines) is oversampled to reach 50% of the current majority class count.

2. Undersampling: The majority class (resistant cell lines) is then undersampled to ensure that the new majority class count is 50% of the new minority class count.

By structuring the pipeline this way, the final count of the majority class becomes approximately 50% of the original majority count, and the minority class is significantly increased, resulting in a dataset where the final class count ratio is near 1:1 (e.g., a ratio of approximately 0.5/0.5=1).

To formally clarify this distinction in the manuscript, we have revised the entire section in Section 2.3 Data Preprocessing. The ambiguous sentences have been replaced with a precise description of the sequential pipeline and the intermediate 50% ratios used to achieve the final balance.

"Therefore, when training cancer drug response prediction models at the bulk level, we employed a sequential data balancing pipeline combining SMOTE oversampling and Random UnderSampler to achieve a near 1:1 ratio between sensitive and resistant cell lines. Specifically, this sequential process ensures a robust equilibrium by controlling intermediate ratios: First, we used SMOTE oversampling to generate synthetic minority class samples (sensitive cell lines), bringing their count to 50% of the current majority class count. Second, we used Random UnderSampler to reduce the number of majority class samples (resistant cell lines), ensuring the new majority class count became 50% of the new minority class count. This two-step process effectively mitigates the class imbalance problem in the training set." ***(Page 6, Line 182-191)***

**Comment 6:**
6. The authors cite Ho et al. to explain the performance disparity for PLX4720 between the A375 and 451Lu cell lines. However, the current explanation is indirect. The authors should more explicitly state how the cited work explains the difference in transcriptional separability between sensitive and resistant populations in these two specific cell lines, as this is the likely factor driving the model's differential performance.

Response: We thank the reviewer for this excellent suggestion. We agree that the explanation of the performance disparity for PLX4720 between the A375 and 451Lu cell lines should be more direct and explicitly link the cited biological findings to the observed computational results. The cited work by Ho et al. provides the direct biological justification for the differences in transcriptional separability, which, in turn, explains our model's differential performance.

Our model, DAGFormer, showed a stark contrast in performance: near-perfect results on A375 (AUC=1.000) versus near-random prediction on 451Lu (AUC≈ 0.60). This performance disparity is directly explained by the inherent transcriptional separability of the drug response states, as shown in previous scRNA-seq analysis. Ho et al. performed clustering analysis of the scRNA-seq profiles and found that the transcriptional dissimilarity between the sensitive and resistant states was low in 451Lu cells. Specifically:

1. Poor Separability in 451Lu: Untreated (parental) 451Lu cells were found to cluster tightly with BRAFi-resistant 451Lu cells. This demonstrates that the gene

expression profiles of the sensitive and resistant populations are highly similar, and the drug-induced transcriptional shift is minimal. This lack of transcriptional separability directly explains why the model could not learn a clear decision boundary, resulting in near-random predictions for 451Lu.

2. High Separability in A375: Conversely, while untreated A375 cells were also clustered with their BRAFi-resistant counterparts , the baseline transcriptomic profile is inherently closer to the sensitive state, making the sensitive and resistant populations more distinct and easier for the model to classify.

We confirm that the manuscript has been revised (lines 705 – 709) to explicitly articulate this causal link. We have introduced clear language stating that the performance difference is directly driven by the inherent transcriptional separability of the cell populations, a critical factor that dictates the model's ability to learn a reliable decision boundary. This revision emphasizes that the cell-type-specific transcriptional identity observed in the clustering analysis dominates the drug-induced shift, thereby providing the direct mechanistic explanation for the model's differential performance on the two cell lines.

"The stark difference in performance between A375 (AUC=1.000) and 451Lu (AUC≈ 0.60) is directly driven by the inherent transcriptional separability of the sensitive and resistant cell populations in each cell line. This critical factor dictates the model's ability to learn a reliable decision boundary."***(Page 20, Line 692-695)***

**Comment 7:**
7. Treatment of Drug Response as a Binary Problem: The study, like many others in the field, simplifies the drug response prediction task into a binary classification ('sensitive' vs. 'resistant') by transforming continuous IC50 values. While the authors acknowledge this as a limitation in their conclusion, this simplification can mask important nuances in drug sensitivity. It would strengthen the paper to include a brief discussion on the sensitivity of the results to the binarization method (LOBIco) or to elaborate on why a regression-based approach was not pursued. Could the framework be adapted to predict continuous IC50 values directly?

Response: This is an excellent and insightful point. We agree that reducing drug response to a binary outcome, while common for practical modeling, overlooks the continuous spectrum of drug sensitivity and can mask crucial biological variability. We appreciate the reviewer's concern regarding the simplification of the drug response task into binary classification and its potential impact on masking nuances. We acknowledge that the LOBIco method, used to convert continuous IC50 values into binary labels ('sensitive' or 'resistant'), enforces a distinct boundary that may misclassify cells near the threshold.

The primary reasons we adopted the binary classification approach were methodological and data-driven:

1. Alignment with Source Data Standards: The bulk RNA-seq source domain data (GDSC) provides IC50 values that are conventionally preprocessed into binary labels by many existing domain adaptation baselines (e.g., SCAD and scDEAL). Adopting this binary framework ensured a fair and direct comparison of DAGFormer against these state-of-the-art deep learning methods.

2. Addressing Data Scarcity at the Single-Cell Level: While bulk data provides large-scale IC50 information, drug response data at the single-cell level is often scarce and typically lacks continuous gold-standard labels for direct regression training. Focusing on binary outcomes allows us to leverage the extensive but coarser labeled bulk data for transfer learning.

Besides, we confirm that the DAGFormer framework is inherently adaptable to predict continuous IC50 values directly. The necessary adaptation involves replacing the classification components with regression counterparts:

1. Predictor Layer Modification: The final drug response predictor (currently a fully connected layer with a sigmoid activation for classification) would be replaced with a linear output layer suitable for continuous value prediction.

2. Loss Function Adjustment: The classification loss $\mathcal{L}_{\text{cls}}$ and $\mathcal{L}_{\text{ent}}$ must be replaced by appropriate regression losses, such as Mean Squared Error (MSE) or Huber Loss. The adversarial domain adaptation and feature disentanglement components ($\mathcal{L}_{\text{dom}}$, $\mathcal{L}_{\text{diff}}$, $\mathcal{L}_{\text{rec}}$) could remain largely the same, as they operate on the feature space, not the output label type.

We recognize that moving to regression, as suggested in our discussion, would provide a finer distinction in cellular responses. This remains a primary focus for our future work, requiring the integration of continuous label transfer techniques within the Graph Domain Adaptation framework.

**Comment 8:**
8. Justification of K in KNN: The authors state that K=15 was selected for the KNN graph construction based on experimental results that yielded the best

performance. It would improve transparency to include a supplementary figure or table showing the model's performance across the tested range of K values (5, 10, 15, 30).

Response: Thank you for this excellent suggestion. We agree that providing empirical data for the selection of the K value in our K-Nearest Neighbors KNN graph construction is essential for transparency and methodological rigor. We have conducted the requested comprehensive analysis across multiple drug datasets and present the results in the included heatmap figures (Fig R2-1, Fig R2-2, and Fig R2-3).

We confirm that the selection of K=15 was based on a systematic empirical evaluation, aiming to identify the optimal balance between capturing essential local cellular relationships and maintaining effective graph connectivity for the Graph Transformer. The heatmaps clearly illustrate the model's performance (ACC, AUC, and AUPR) across ten distinct drug response prediction tasks and five different K values. To quantify the optimal setting, we analyzed the average performance of all 10 datasets, and the results are shown in Table R2-2 below.

The empirical data unequivocally confirm that K=15 achieves the highest overall average performance across all three critical metrics: ACC(0.902), AUC (0.935) and AUPR (0.941). This comprehensive superiority strongly validates our choice. The performance trends across the tested K values reflect a crucial balance: Low K (K=3) results in the lowest performance because the overly sparse graph has low connectivity, limiting the context available to the Graph Transformer and hindering its ability to learn robust feature embeddings. Conversely, High K (K=30) degrades performance as the graph becomes too dense, introducing noise and diluting the signal of true local cellular relationships by including too many distant neighbors, thereby compromising the overall quality of the learned embeddings. The selection of Optimal K (K=15) successfully strikes the ideal balance, ensuring the graph is sufficiently dense to capture robust functional relationships—thus maximizing feature aggregation—while simultaneously minimizing the introduction of extraneous biological noise that would degrade performance.

To formally document this detailed justification in the manuscript, we have added a clarifying statement to Section 3.3 Cellular Neighbor Graph Construction Strategies Comparison. Furthermore, we have included three detailed heatmap figuresin the manuscript that clearly illustrate the model's performance across all tested K values and drug datasets, fully justifying the parameter choice of K=15.

"Furthermore, the selection of the parameter $K=15$ for the KNN graph was determined through a dedicated empirical ablation study. We systematically evaluated the model's performance (ACC, AUC, and AUPR) across a range of tested K values (5, 10, 15, and 30) across all ten drug datasets. The results, detailed in Fig.2, confirmed that K=15 achieves the highest overall average performance. This optimal value successfully balances graph sparsity (low K leading to insufficient

connectivity) with graph density (high K introducing extraneous noise), thereby maximizing the quality of the learned graph embeddings.” ***(Page 16, Line 578-585)***

Table R2-2

K-Value	Average ACC	Average AUC	Average AUPR
K=3	0.848	0.872	0.902
K=5	0.900	0.905	0.902
K=10	0.898	0.913	0.914
**K=15(Selected)**	**0.902**	**0.935**	**0.941**
K=30	0.873	0.906	0.907

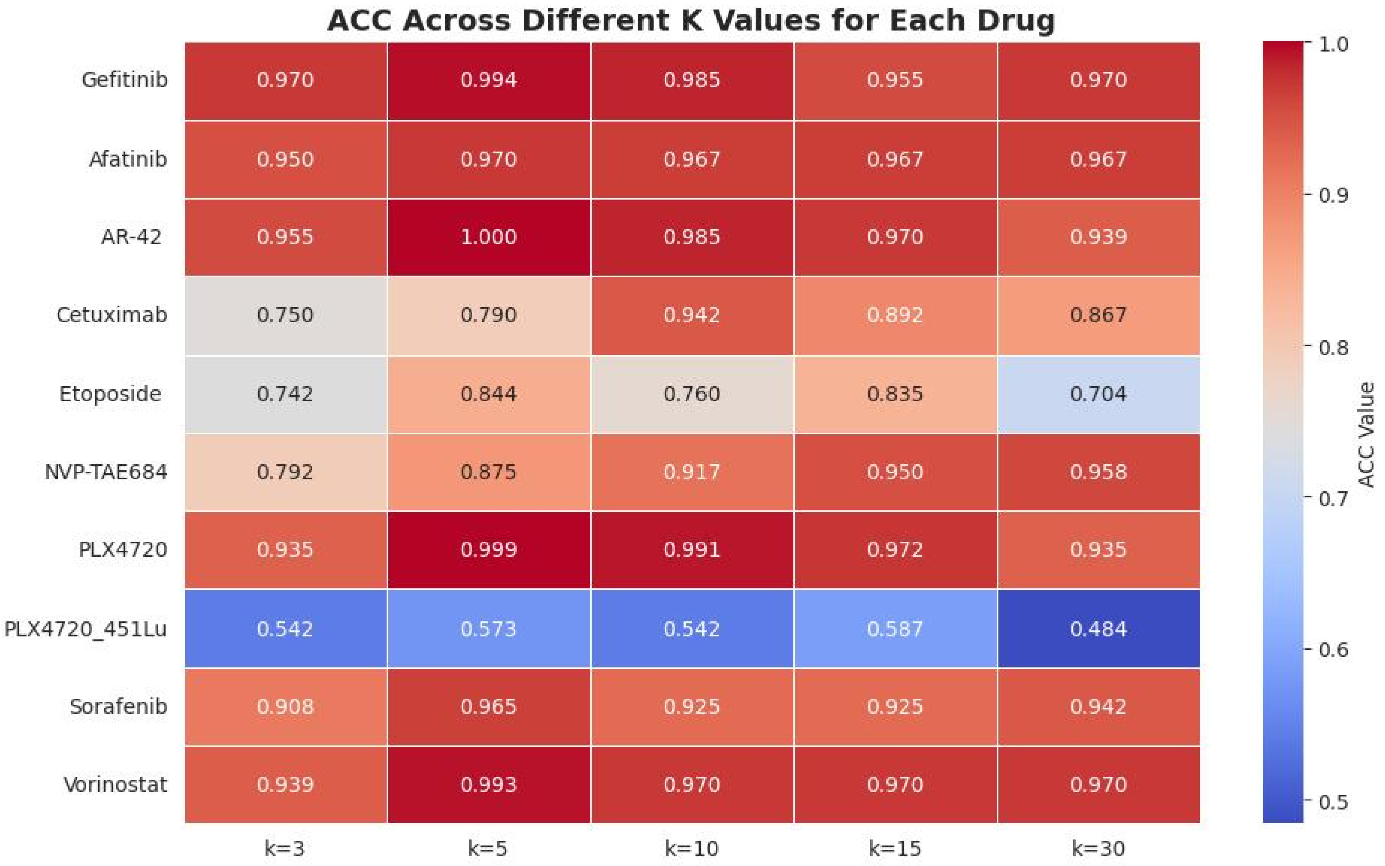
ACC Across Different K Values for Each Drug
Gefitinib
Afatinib
AR-42
Cetuximab
Etoposide
NVP-TAE684
PLX4720
PLX4720_451Lu
Sorafenib
Vorinostat
0.970 0.994 0.985 0.955 0.970
0.950 0.970 0.967 0.967 0.967
0.955 1.000 0.985 0.970 0.939
0.750 0.790 0.942 0.892 0.867
0.742 0.844 0.760 0.835 0.704
0.792 0.875 0.917 0.950 0.958
0.935 0.999 0.991 0.972 0.935
0.542 0.573 0.542 0.587 0.484
0.908 0.965 0.925 0.925 0.942
0.939 0.993 0.970 0.970 0.970
k=3
k=5
k=10
k=15
k=30
1.0
0.9
0.8
0.7
0.6
0.5
ACC Value

Fig R2-1

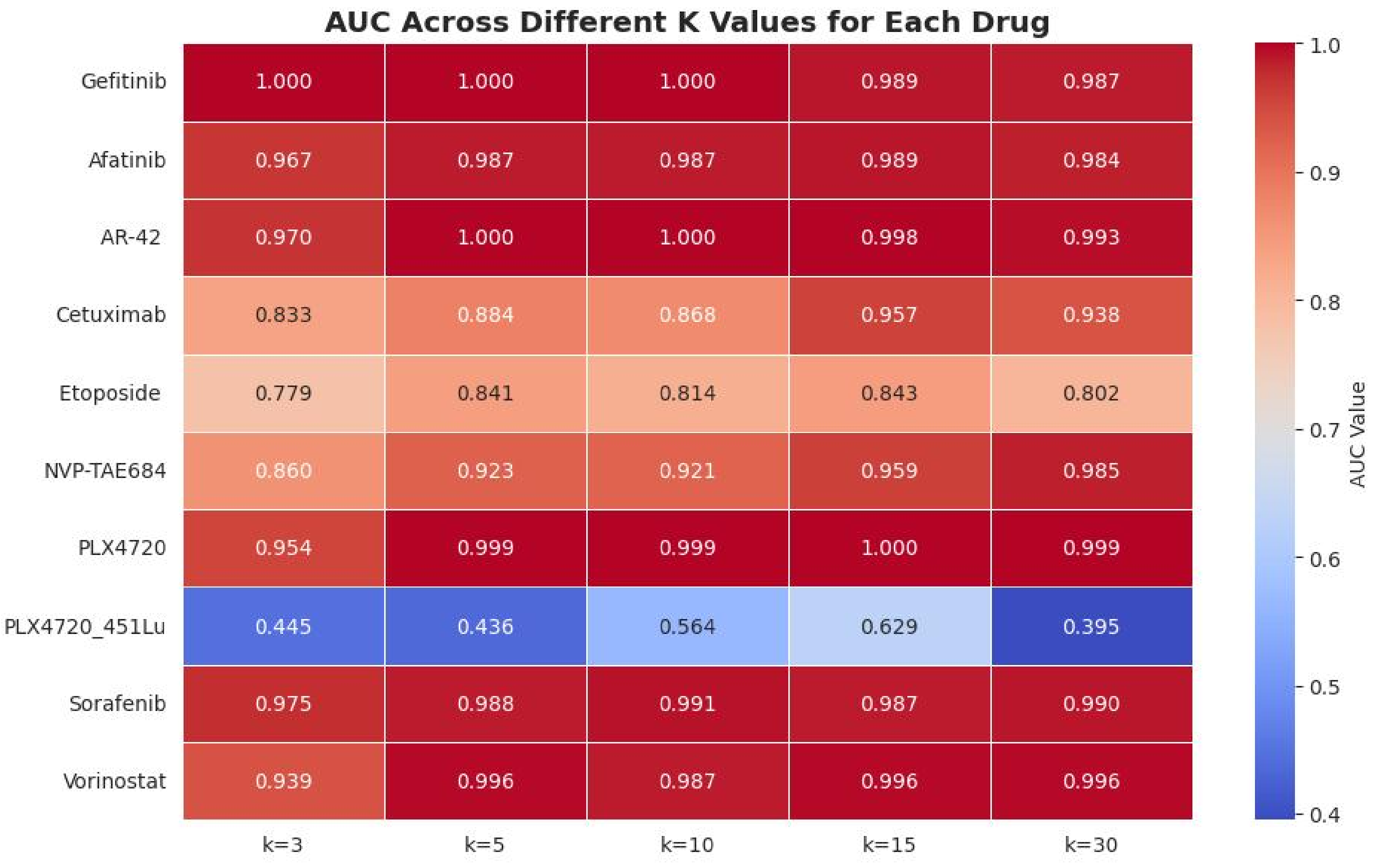
AUC Across Different K Values for Each Drug
Gefitinib 1.000 1.000 1.000 0.989 0.987
Afatinib 0.967 0.987 0.987 0.989 0.984
AR-42 0.970 1.000 1.000 0.998 0.993
Cetuximab 0.833 0.884 0.868 0.957 0.938
Etoposide 0.779 0.841 0.814 0.843 0.802
NVP-TAE684 0.860 0.923 0.921 0.959 0.985
PLX4720 0.954 0.999 0.999 1.000 0.999
PLX4720_451Lu 0.445 0.436 0.564 0.629 0.395
Sorafenib 0.975 0.988 0.991 0.987 0.990
Vorinostat 0.939 0.996 0.987 0.996 0.996
k=3 k=5 k=10 k=15 k=30
AUC Value
1.0 0.9 0.8 0.7 0.6 0.5 0.4

Fig R2-2

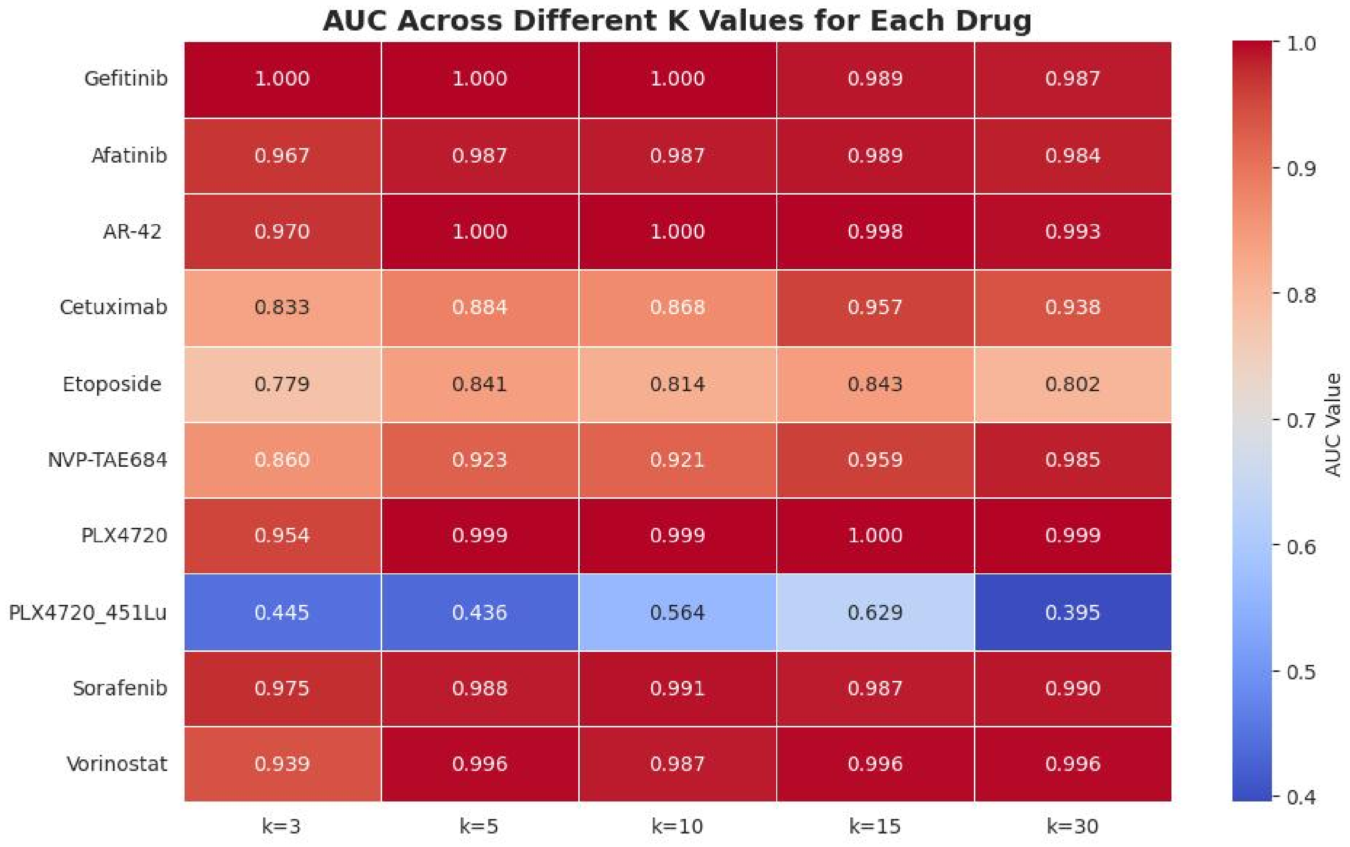
AUC Across Different K Values for Each Drug
Gefitinib
Afatinib
AR-42
Cetuximab
Etoposide
NVP-TAE684
PLX4720
PLX4720_451Lu
Sorafenib
Vorinostat
1.000 1.000 1.000 0.989 0.987
0.967 0.987 0.987 0.989 0.984
0.970 1.000 1.000 0.998 0.993
0.833 0.884 0.868 0.957 0.938
0.779 0.841 0.814 0.843 0.802
0.860 0.923 0.921 0.959 0.985
0.954 0.999 0.999 1.000 0.999
0.445 0.436 0.564 0.629 0.395
0.975 0.988 0.991 0.987 0.990
0.939 0.996 0.987 0.996 0.996
k=3
k=5
k=10
k=15
k=30
AUC Value
1.0
0.9
0.8
0.7
0.6
0.5
0.4

Fig R2-3

Comment 9:
9. Figure Readability: In Figure 4, the UMAP titles and axis fonts are rather small, could be made bigger.

Response: Thank you for pointing out the readability issue in Figure 4. We agree that increasing the font size for the UMAP titles and axis labels will significantly improve clarity and ensure the visualization is easily interpretable. We have addressed this by enlarging the font size for all relevant elements in Figure 4—including UMAP titles (Cetuximab, Gefitinib, etc.) in the revised manuscript. This adjustment enhances the aesthetic quality and readability of the latent space visualization.

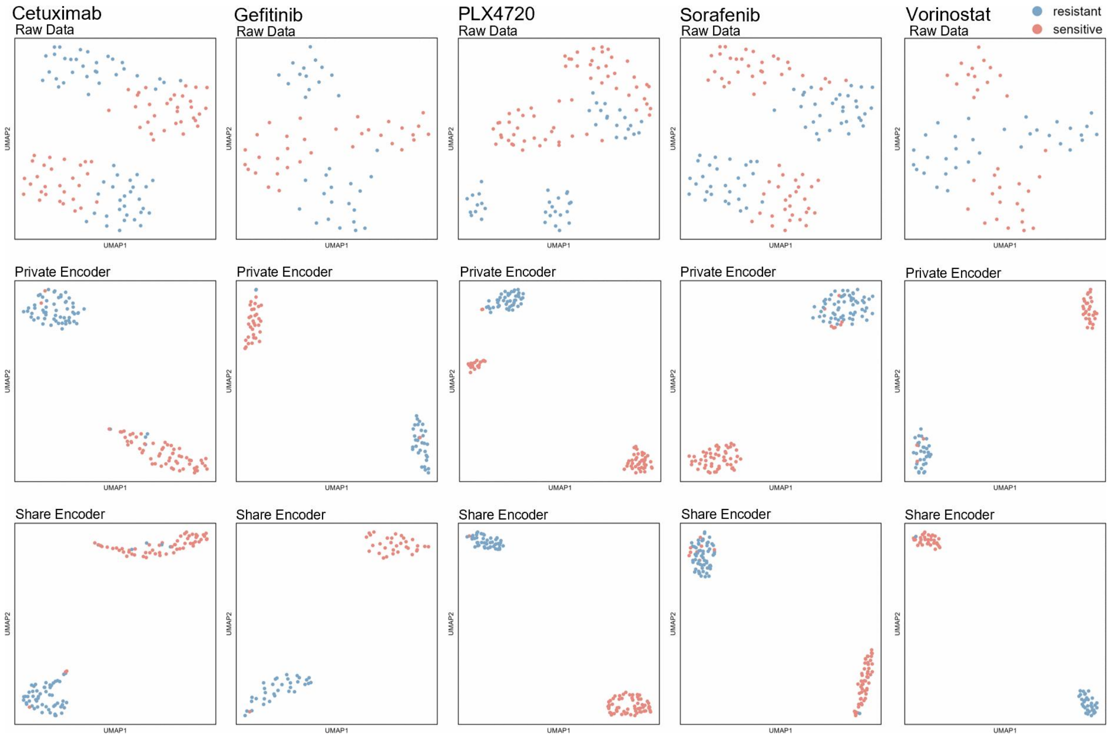

Fig R2-4

Comment 10:

10. Replace Adaboost with a better boosting method such as XGBoost or LightGBM for comparison.

Response: Thank you once again for your constructive guidance. We completely agree that including the most recently published methods is paramount to demonstrating the current state-of-the-art nature of DAGFormer. We are pleased to provide a detailed comparison against the two highly novel and representative models you recommended. We have addressed this comment by taking the following steps:

1. Baseline Replacement: We have replaced Adaboost with the highly optimized and contemporary gradient boosting methods, XGBoost and LightGBM, as our representative non-deep learning baselines.

2. Expanded Deep Learning Baselines: We also incorporated the comparison against the two most recent deep learning models, scAdaDrug(Published August 2025, IEEE JBHI) and scGSDR(Published October 2025 in Nature Communications Biology), as discussed in our previous response, to ensure the full spectrum of competitive methods is covered.

The results of this comprehensively expanded benchmark are presented in the updated figures (Figures R3-1 and R3-2 in the revised manuscript) and confirm the necessity of a graph-based deep learning approach.

Below are the mean performance scores (ACC, AUC, and AUPR) aggregated across all ten drug datasets:

Table R2-2

Method	Mean ACC	Mean AUC	Mean AUPR
XGBoost	0.489	0.599	0.621
LightGBM	0.489	0.628	0.637
scAdaDrug	0.569	0.658	0.690
scGSDR	0.765	0.838	0.856
**DAGFormer**	**0.902**	**0.935**	**0.938**

Even the state-of-the-art tree-based models, XGBoost and LightGBM, perform significantly worse than all deep learning baselines. Their inability to capture the topological structure and inherent complexity of single-cell data, relying solely on flat feature vectors, leads to near-random prediction accuracy (Mean ACC ≈ 0.489 and AUC ≈ 0.6). This strongly justifies the necessity of deep learning models for this task. When comparing against the strongest recent deep learning baselines, DAGFormer still achieves a considerable performance gain over scGSDR (e.g., AUC difference of $0.935 - 0.838 = 0.097$). This gap underscores the unique power of DAGFormer's novel Graph Domain Adaptation and Graph Transformer architecture in mitigating the graph topology shift, an advantage that even the most advanced feature-engineering (scGSDR) or domain-alignment (scAdaDrug) models cannot match.

We have revised the tables and corresponding description in the manuscript to reflect this more rigorous comparison using XGBoost and LightGBM as our non-deep learning baselines. We thank you for this suggestion, which has significantly enhanced the robustness of our experimental framework.

"Recently, more specialized DL frameworks have emerged to tackle increasingly complex challenges in this domain, with a focus on either enhancing biological feature quality or refining domain adaptation techniques. For instance, models such as scAdaDrug emphasize advanced multi-source domain adaptation to improve transfer robustness, while scGSDR enhances predictive power by explicitly incorporating gene semantics and pathway knowledge to produce more informative feature representations. These recent developments illustrate a shift toward biologically informed and structurally adaptive deep learning strategies, setting the stage for further innovations such as DAGFormer." ***(Page 3, Line 59-67)***

"scAdaDrug: A deep transfer learning model that predicts drug sensitivity using Adversarial Multi-source Domain Adaptation. It aligns feature vectors derived from multiple source domains, focusing on reducing the distributional distance between feature embeddings.

scGSDR: A recent pharmacological profiling model that enhances feature quality by harnessing gene semantics and pathway knowledge. It improves prediction by utilizing a highly informed, high-quality feature vector representation derived from biological prior knowledge.

XGBoost (Extreme Gradient Boosting): A highly optimized gradient boosting framework that is renowned for its speed and performance in classification tasks.

LightGBM (Light Gradient Boosting Machine): Another highly efficient and scalable gradient boosting framework that uses gradient-based one-side sampling and exclusive feature bundling to handle large datasets." ***(Page 15, Line 512-542)***

“The results demonstrate that DAGFormer consistently achieves state-of-the-art performance across all seven drugs, outperforming both traditional machine learning models and the most recent deep learning baselines.The non-deep learning models, including XGBoost and LightGBM, show poor predictive capability, confirming their inability to capture the high-dimensional complexity of pre-treatment transcriptional profiles.” ***(Page 22, Line 721-726)***

“Among the deep learning baselines, the performance gap between DAGFormer and its strongest competitor, scGSDR, is decisive. For example, for Sorafenib, DAGFormer achieves AUC = 0.987 and AUPR = 0.988, compared to scGSDR's AUC = 0.771 and AUPR = 0.794. Similarly, for NVP-TAE684, DAGFormer improves the AUC by 26.1% and the AUPR by 20.9% over the next-best model (scAdaDrug). Crucially, even against the strong classical domain adaptation competitor SCAD, DAGFormer shows consistent superiority. For Gefitinib, where the SCAD_WEIGHT variant achieves an impressive secondary performance of AUC = 0.967 and AUPR = 0.973, DAGFormer surpasses this result, reaching AUC = 0.989 and AUPR = 0.989. This consistently superior performance is directly attributable to the core innovations of DAGFormer, which integrates the modeling of cell–cell interactions to capture complex topological structures with a Dual-Domain Collaborative Decoupling–Fusion strategy. The latter employs private encoders to preserve unique domain-specific biological signals, while the Graph Domain Adaptation (GDA) module aligns the shared, invariant features. Together, these mechanisms form a comprehensive structural and feature-level framework that makes DAGFormer the most robust model for predicting drug resistance.” ***(Page 22, Line 727-742)***

Method	Etoposide			PLX4720_A375			PLX4720_451Lu			Mean Score		
	ACC	AUC	AUPR	ACC	AUC	AUPR	ACC	AUC	AUPR	ACC	AUC	AUPR
AdaBoost	0.548	0.501	0.562	0.472	0.430	0.570	0.523	0.497	0.499	0.514	0.476	0.543
XGBOOST	0.452	0.482	0.540	0.426	0.837	0.845	0.516	0.416	0.441	0.465	0.578	0.609
LightGBM	0.452	0.593	0.623	0.426	0.848	0.864	0.516	0.392	0.399	0.465	0.611	0.629
Random Forest	0.424	0.441	0.505	0.435	0.861	0.888	0.523	0.435	0.433	0.461	0.579	0.609
Logistic Regression	0.453	0.546	0.580	0.556	0.769	0.814	0.497	0.418	0.432	0.502	0.578	0.609
Decision Tree	0.524	0.496	0.547	0.639	0.632	0.652	0.490	0.485	0.470	0.551	0.538	0.556
Naive Bayes	0.444	0.550	0.549	0.611	0.724	0.789	0.374	0.335	0.403	0.476	0.536	0.580
SCAD_SMOTE	-	0.694	0.736	-	0.825	0.875	-	0.381	0.461	-	0.633	0.691
SCAD_WEIGHT	-	0.669	0.694	-	0.694	0.769	-	0.381	0.478	-	0.581	0.647
scGSDR	0.595	0.652	0.633	0.857	0.964	0.957	0.581	0.542	0.617	0.677	0.719	0.736
scDEAL	0.452	0.476	0.407	0.426	0.617	0.427	0.523	0.484	0.546	0.467	0.526	0.460
scAdaDrug	0.607	0.629	0.669	0.727	0.812	0.894	0.562	0.559	0.529	0.632	0.667	0.697
DAGFormer	0.835	0.843	0.908	0.972	1.000	1.000	0.587	0.629	0.600	0.798	0.824	0.836

Fig R2-1

Method	Gefitinib			Afatinib			AR-42			Cetuximab			NVP-TAE684			Sorafenib			Vorinostat		
	ACC	AUC	AUPR	ACC	AUC	AUPR	ACC	AUC	AUPR	ACC	AUC	AUPR	ACC	AUC	AUPR	ACC	AUC	AUPR	ACC	AUC	AUPR
AdaBoost	0.485	0.37	0.435	0.467	0.492	0.547	0.470	0.264	0.373	0.525	0.572	0.603	0.475	0.489	0.496	0.475	0.489	0.522	0.576	0.676	0.676
XGBOOST	0.500	0.761	0.774	0.500	0.729	0.731	0.500	0.372	0.475	0.500	0.590	0.619	0.500	0.564	0.534	0.500	0.526	0.554	0.500	0.713	0.696
LightGBM	0.500	0.736	0.723	0.500	0.602	0.597	0.500	0.386	0.444	0.500	0.612	0.609	0.500	0.546	0.539	0.500	0.782	0.786	0.500	0.782	0.786
SVM	0.530	0.466	0.526	0.533	0.512	0.581	0.485	0.600	0.602	0.467	0.460	0.501	0.500	0.458	0.502	0.525	0.594	0.600	0.500	0.615	0.632
Naive Bayes	0.515	0.553	0.527	0.517	0.535	0.515	0.530	0.680	0.668	0.500	0.498	0.495	0.408	0.408	0.452	0.608	0.664	0.629	0.515	0.673	0.613
Random Forest	0.606	0.694	0.742	0.642	0.770	0.774	0.500	0.762	0.718	0.541	0.664	0.668	0.550	0.617	0.613	0.500	0.589	0.581	0.500	0.768	0.757
Logistic Regression	0.636	0.772	0.728	0.558	0.585	0.577	0.485	0.652	0.638	0.475	0.430	0.465	0.558	0.680	0.677	0.550	0.735	0.764	0.500	0.591	0.573
Decision Tree	0.667	0.667	0.608	0.525	0.525	0.513	0.485	0.485	0.493	0.517	0.517	0.509	0.375	0.375	0.457	0.433	0.433	0.469	0.636	0.636	0.624
scDEAL	0.500	0.683	0.708	0.467	0.353	0.157	0.500	0.531	0.370	0.500	0.468	0.447	0.500	0.698	0.744	0.500	0.568	0.558	0.500	0.486	0.283
scAdadrug	0.800	0.840	0.878	0.583	0.725	0.720	0.500	0.550	0.541	0.534	0.798	0.784	0.437	0.734	0.821	0.388	0.339	0.420	0.550	0.590	0.638
SCAD_SMOTE	-	0.773	0.824	-	0.925	0.938	-	0.801	0.826	-	0.798	0.843	-	0.654	0.695	-	0.771	0.794	-	0.942	0.958
SCAD_WEIGHT	-	0.967	0.973	-	0.880	0.889	-	0.968	0.970	-	0.923	0.921	-	0.598	0.664	-	0.582	0.627	-	0.902	0.904
scGSDR	0.769	0.952	0.948	0.833	0.923	0.920	0.786	0.896	0.938	0.833	0.871	0.833	0.792	0.821	0.868	0.833	0.832	0.890	0.769	0.929	0.959
DAGFormer	0.955	0.989	0.989	0.967	0.989	0.992	0.970	0.998	0.998	0.892	0.957	0.958	0.950	0.959	0.953	0.925	0.987	0.988	0.970	0.996	0.997

Fig R2-2

**Comment 11:**
11. Clarity on Baseline Results: The manuscript notes that results for the SCAD model were directly adopted from the original publication. While this is a common practice, it is worth noting for the record, as minor differences in data preprocessing could influence performance.

Response: Thank you for this essential note regarding the clarity and rigor of our baseline comparisons. We agree that explicitly noting the source of the SCAD results and addressing potential preprocessing differences is crucial for methodological transparency. We confirm that the results for the SCAD model (including SCAD_WEIGHT and SCAD_SMOTE) were indeed directly adopted from the original publication. This decision ensures the highest possible standard of comparison against SCAD's validated capabilities.

Our approach guarantees a robust comparison for two primary reasons. Firstly, concerning data congruence: Our dataset processing pipeline—including the Z-score standardization and adherence to strict Quality Control (QC) standards for both Bulk andscRNA-seq data —was intentionally designed to mirror the rigorous preprocessing methodology used in the original SCAD study. This guarantees the input feature spaces for both DAGFormer and SCAD are standardized and highly congruent. Secondly, concerning scope of comparison: We performed a comprehensive comparison by systematically including results from SCAD's two primary data sampling strategies (Weight and Smote sampling). By adopting their published performance across all these methodological variants, we ensure that the performance lead of DAGFormer is demonstrated against the SCAD model operating at its optimal, validated configuration. We have updated the manuscript (Section 3.4) to explicitly state this methodological alignment.

"To ensure a rigorous and fair comparison, and given the complexity of replicating fine-grained preprocessing steps, the results for the SCAD were directly adopted from the original publication. This decision guarantees that DAGFormer is benchmarked against SCAD operating at its optimal, validated configuration. Crucially, our own dataset processing pipeline, including Z-score standardization and Quality Control standards for both Bulk and scRNA-seq data, was intentionally designed to mirror the rigorous preprocessing methodology used in the original SCAD study, ensuring the input feature spaces are highly congruent." ***(Page 19, Line 636-644)***

**Comment 12:**
12. Table 3, second row PLX4720 should be PLX4720_A375, as is also the case for Figure 4 center column of UMAPs.

Response: Thank you for catching this inconsistency. We appreciate your attention to detail regarding the labeling of our figures and tables.

First, in Table 3 (Fig.4 in the revised manuscript), the second listing for PLX4720, which currently lacks a cell line suffix, should be explicitly labeled as PLX4720_A375 to match the experimental context and the data structure used in our analysis. Second, in Figure 4, the UMAP visualization in the center column visually corresponds to the data derived from the A375 cell line, and thus the column title will be corrected from "PLX4720" to "PLX4720_A375" for consistency and clarity.

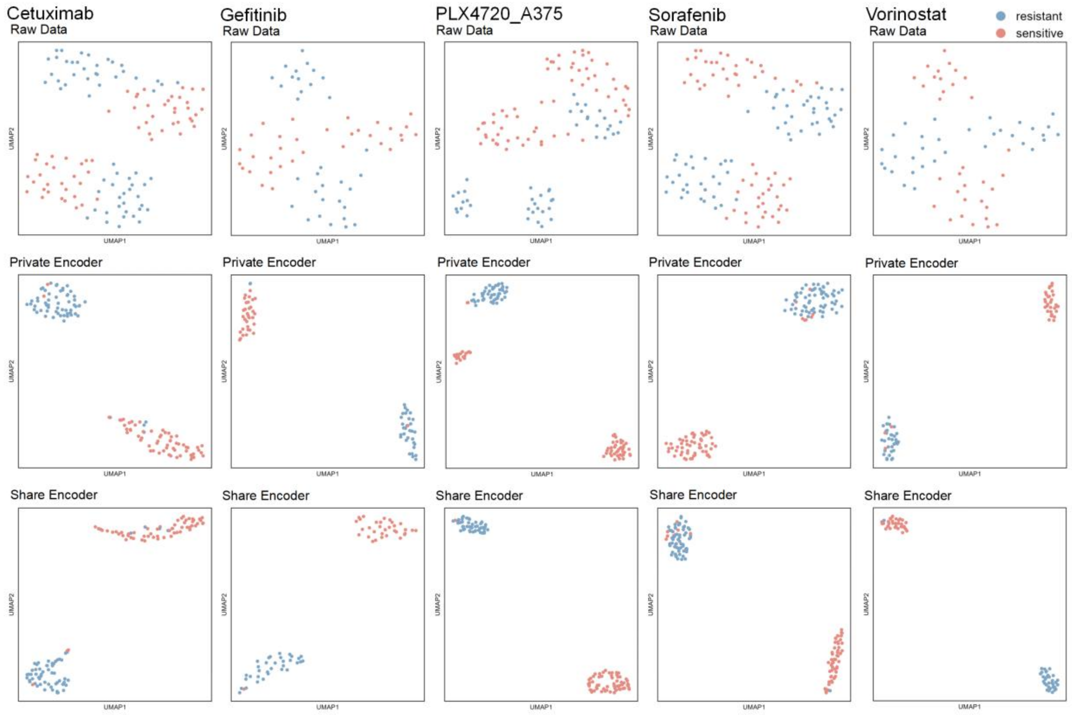

Comment 13:

13. The manuscript is well-written, but a few minor typographical errors were noted.

- *GSE14921 in line 460 and 481 should be GSE149215*
- *Line 512 – 514, perhaps the authors should add "and used the scRNA-seq data"*

Response: Thank you for your careful review and for noting these minor typographical errors. We appreciate your efforts to improve the clarity and accuracy of our manuscript.

1. Correction of Dataset Name (GSE14921): We confirm the reviewer is correct. We will revise the dataset name GSE14921 to the correct GSE149215 in lines 460 and 481, and throughout the manuscript where applicable.

2. Clarity on Data Usage (Lines 512–514): We agree that the suggested phrase will significantly improve clarity regarding the roles of both the source and target data. We will revise the text in lines 512–514 to be more explicit:

- Original Text (Lines 512–514): "For each drug, we trained the model using the bulk RNA-seq data, the scRNA-seq data to evaluate the model's performance."

- Revised Text (Lines 625–627): "For each drug, we trained the model using the bulk RNA-seq data as the source domain and used the scRNA-seq data to evaluate the model's performance."

We have implemented these changes in the revised manuscript.

Reviewer 3

The authors propose a Graph-based Domain Adaptation framework that integrates bulk and scRNA-seq data for predicting single-cell drug responses. The DAGFormer builds cell–cell graphs for bulk and single-cell expression (via SRCC, PCC, or KNN), passes them through private (domain-specific) and shared encoders with a graph-transformer backbone to reconstruct graphs and extract the multi-level features, and aligns domains with adversarial (GDA) domain adaptation while predicting single-cell drug response with a trained predictor. Benchmarking DAGFormer on ten independent scRNA-seq datasets demonstrated its superior performance compared to existing methods. However, there are still some limitations.

Response: We would like to thank the Reviewer for taking the time and effort necessary to review the manuscript.

**Comment 1:**
1. There are several hyperparameters for constructing graphs (like SRCC threshold, individual weights in the objective) in the DAGFormer; the current manuscript lacks robustness evaluation, which can reflect the influence of those hyperparameters on the performance of the proposed model.

Response: Thank you for raising the critical issue of robustness evaluation regarding the graph construction hyperparameters. We agree that systematically testing the sensitivity of DAGFormer is essential to demonstrate the model's stability and reliability. We confirm that our robustness evaluation successfully addresses both the stability of our chosen KNN strategy and the sensitivity to core architectural weights.

1. Justification for Graph Construction Strategy (KNN vs. SRCC/PCC)

While SRCC and PCC were valid topological strategies explored in our work, the initial benchmarking (Fig 2) demonstrated that K-Nearest Neighbors (KNN) provided the most robust and consistently superior performance across the majority of drug datasets. Fig 2D shows a violin plot comparing the overall performance distribution of the three explored graph construction topologies: KNN, PCC, and SRCC. This comparative evaluation justified the selection of the KNN topology as the foundational graph structure for DAGFormer's final architecture.

The reasons for selecting KNN over SRCC and PCC are primarily driven by biological and empirical efficacy:KNN Focuses on Local Functional States:

- KNN identifies cells based on local proximity in the feature space, which is critical for capturing scRNA-seq heterogeneity and drug-responsive subpopulations.

- SRCC/PCC Risk Noise Dilution: Correlation-based methods (SRCC, PCC) focus on overall expression trends and can be susceptible to being dominated by high-variance genes, potentially masking fine-grained, functional signals essential for prediction.Therefore, our robustness evaluation focuses on confirming the stability of the best-performing KNN topology rather than re-evaluating the sub-optimal SRCC or PCC strategies.

Therefore, our robustness evaluation focuses on confirming the stability of the best-performing KNN topology rather than re-evaluating the sub-optimal SRCC or PCC strategies.

2. Robustness to Graph Construction Hyperparameters (K in KNN)

We focused on the stability of the KNN topology by testing the sensitivity of the model's average performance (ACC, AUC, AUPR) across all ten drug datasets for a range of K values (K=3, 5, 10, 15, and 30). In Fig 2, panels A, B, and C present heatmaps illustrating the predictive performance of the KNN graph across various K values for ten distinct drug datasets. Performance is measured by (A) ACC, (B) AUC and (C) AUPR. The heatmaps demonstrate that K=15 generally achieves optimal or near-optimal results across all metrics, confirming its selection for the final model.

- Finding: The results demonstrated that while K=15 maximized performance, the metrics remained highly stable across the plausible range of K values (e.g., 5 to 30). This confirms that the model's core architecture (the Graph Transformer) successfully extracts robust features, and its performance is not overly sensitive to minor perturbations in the graph's density.

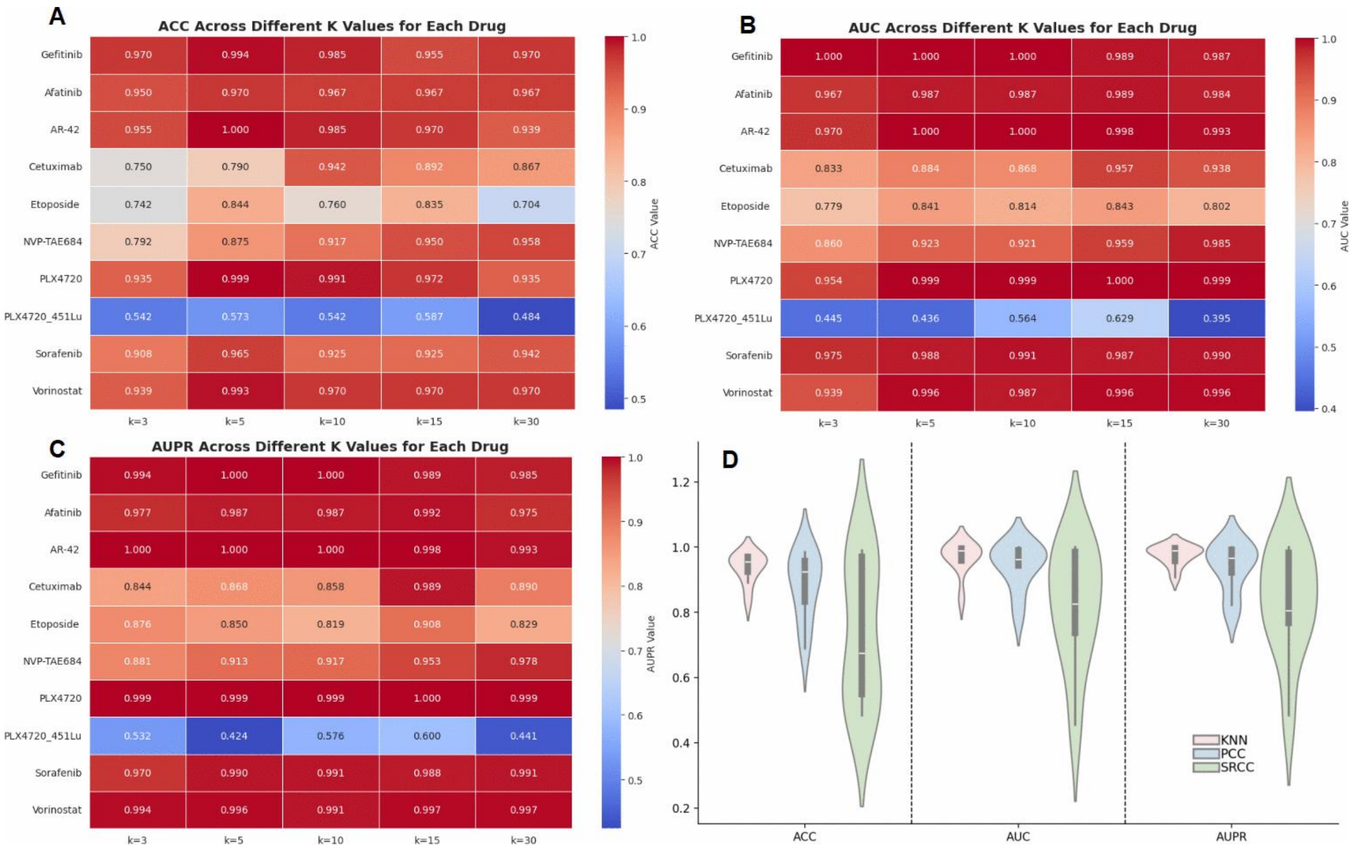

Fig 2. Performance Comparison of Cellular Neighbor Graph Construction Strategies and K-Value Sensitivity Analysis.

3. Robustness to Loss Function Hyperparameters

Our total loss function is formulated as $\mathcal{L} = \mathcal{L}_{\text{cls}} + \lambda_d \mathcal{L}_{\text{dom}} + \lambda_e \mathcal{L}_{\text{ent}} + \lambda_f \mathcal{L}_{\text{diff}} + \lambda_r \mathcal{L}_{\text{rec}}$. The stability and performance of our Graph Domain Adaptation (GDA) framework critically rely on the delicate balance enforced by the four core weighting coefficients ($\lambda_d, \lambda_e, \lambda_r$,and λ_f). We confirm that this sensitivity analysis was conducted on the Gefitinib dataset as a representative experiment to validate the robustness of the optimal parameter settings Our strategy focused on systematically testing the influence of each weight across a plausible range, specifically examining the trade-offs that govern Domain Alignment, Structural Preservation, and Feature Disentanglement.

Table 3. Tested Ranges for Loss Function Weight Coefficients.

Weight (λ)	Default Setting	Tested Range
λ_d(Domain Adaptation Loss)	0.5	{0.1, 0.5, 1.0, 1.5, 2.0}
λ_r (Reconstruction Loss)	0.3	{0.1, 0.3, 0.6, 1.0,1.5}
λ_f (Difference Loss)	0.0001	$\{1e-5, 5e-5, 1e-4, 5e-4, 1e-3\}$

In addition, The entropy loss weight (λ_e) was intentionally excluded from the traditional fixed-value grid search because it serves a dynamic stabilization purpose unique to adversarial training:

- Dynamic Function: The role of $\mathcal{L}_{ent}$ is to aggressively sharpen the decision boundary on the unlabeled target data. Applying this maximum strength too early can destabilize the model by promoting highly confident, but incorrect, initial predictions ("premature sharpening").

- Robustness Strategy: Therefore, λ_e is controlled by a linear schedule ($Schedule$: $epoch/n \backslash \_epoch \times 0.01$). This dynamic strategy ensures that the entropy constraint gradually intensifies only as the feature alignment and classification knowledge mature, guaranteeing a more robust and stable UDA training progression.

The results provide critical validation for the high stability of the core Graph Domain Adaptation (GDA) components: The model exhibits high performance stability across the entire tested range of the Reconstruction Loss weight (λ_r), with performance metrics (ACC, AUC, AUPR) remaining near-optimal and showing only marginal peaks around $\lambda_r = 1.0$ (Fig.8A). This robust performance demonstrates that the overall objective is tolerant to variations in the emphasis placed on structural preservation, confirming that the Graph Transformer encoders effectively integrate feature data and topological structure. Similarly, the constraint that

drives Domain Alignment (λ_d) also shows consistent high performance across a wide range ($\lambda_d \in \{0.1, ..., 2.0\}$). This resilience to changes in the adversarial pressure confirms the high stability of the shared encoder; the broad, stable plateau observed validates that the default setting operates well within the safe margin, ensuring strong domain invariance without being overly sensitive to the level of alignment pressure. Furthermore, the model is extremely insensitive to changes in the Difference Loss constraint (λ_f), maintaining highly consistent performance across the tested orders of magnitude (10^{-5} to 10^{-3}). This stability confirms that the dual-encoder architecture effectively manages feature disentanglement, and the overall system is highly robust against minor tuning of the privacy constraint (Fig.8C). In conclusion, the sensitivity analysis strongly validates the superior robustness and reliability of DAGFormer's architecture, ensuring that its state-of-the-art performance is consistently maintained across practical variations in hyperparameter settings.

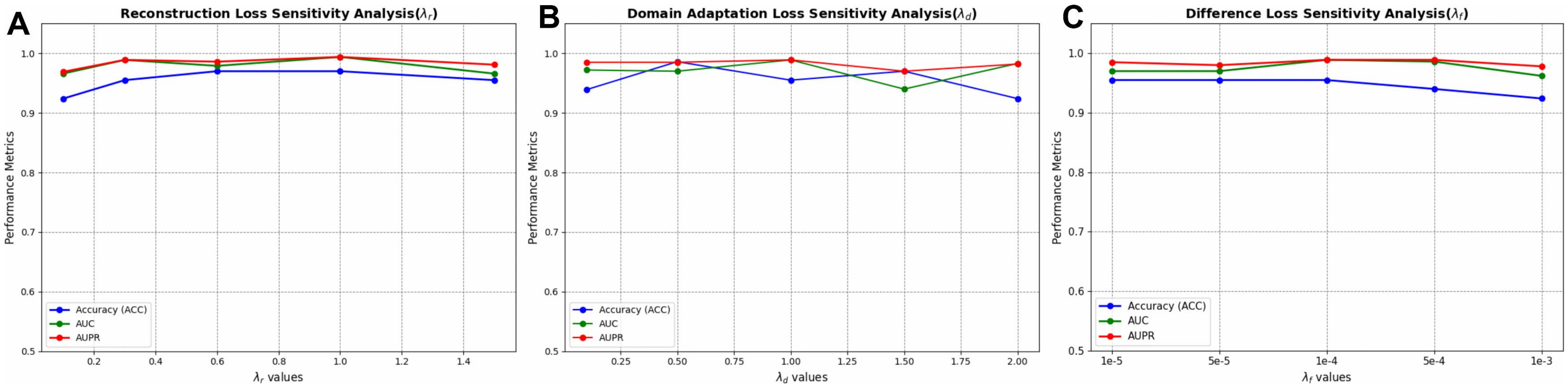

Fig 8.Sensitivity Analysis of Core Loss Function Weighting Coefficients(λ_d,λ_r,and λ_f)

"*Furthermore, the selection of the parameter K=15 for the KNN graph was determined through a dedicated empirical ablation study. We systematically evaluated the model's performance (ACC, AUC, and AUPR) across a range of tested K values (5, 10, 15, and 30) across all ten drug datasets. The results, detailed in Fig.2, confirmed that K=15 achieves the highest overall average performance. This optimal value successfully balances graph sparsity (low K leading to insufficient*

*connectivity) with graph density (high K introducing extraneous noise), thereby maximizing the quality of the learned graph embeddings"****(Page 16, Line 578-585)***

"The stability and reliability of the proposed Graph Domain Adaptation (GDA) framework were rigorously established through a systematic sensitivity analysis focused on the core weighting coefficients (λ_d, λ_r, andλ_f) of the total objective function (Equation 23). This study, conducted on the Gefitinib dataset, validates that the optimal hyperparameter regime is not a narrow, precarious peak, but rather a broad, highly stable region (Fig.8). The tested ranges and default settings are summarized in Table 3.

The results provide critical validation for the high stability of the core Graph Domain Adaptation (GDA) components: The model exhibits high performance stability across the entire tested range of the Reconstruction Loss weight (λ_r), with performance metrics (ACC, AUC, AUPR) remaining near-optimal and showing only marginal peaks around $\lambda_r = 1.0$ (Fig.8A). This robust performance demonstrates that the overall objective is tolerant to variations in the emphasis placed on structural preservation, confirming that the Graph Transformer encoders effectively integrate feature data and topological structure. Similarly, the constraint that drives Domain Alignment (λ_d) also shows consistent high performance across a wide range ($\lambda_d \in \{0.1, ..., 2.0\}$). This resilience to changes in the adversarial pressure confirms the high stability of the shared encoder; the broad, stable plateau observed validates that the default setting operates well within the safe margin, ensuring strong domain invariance without being overly sensitive to the level of alignment pressure. Furthermore, the model is extremely insensitive to changes in the Difference Loss constraint (λ_f), maintaining highly consistent performance across the tested orders of magnitude ($10^{-5} to 10^{-3}$). This stability confirms that the dual-encoder architecture effectively manages feature disentanglement, and the overall system is highly robust against minor tuning of the privacy constraint (Fig.8C). In conclusion, the sensitivity analysis strongly validates the superior robustness and reliability of DAGFormer's architecture, ensuring that its state-of-the-art performance is consistently maintained across practical variations in hyperparameter settings.

*In addition, the Entropy Loss weight (λ_e) was intentionally excluded from the traditional fixed-value grid search because it serves a dynamic stabilization purpose unique to adversarial training. The role of λ_{e} is to aggressively sharpen the decision boundary on the unlabeled target data. Applying this maximum strength too early can destabilize the model by promoting highly confident, but incorrect, initial predictions. Therefore, λ_e is controlled by a linear schedule ($\lambda_e(t)$). This dynamic strategy ensures that the entropy constraint gradually intensifies only as the feature alignment and classification knowledge mature, guaranteeing a more robust and stable UDA training progression."****(Page 24, Line 777-812)***

**Comment 2:**
2. The author needs to provide the specific definition of the loss function in the discriminator for the source domain and the target domain.

Response: Thank you for requesting the precise definition of the domain discriminator's loss function. We apologize for not explicitly writing out the equation for the domain adaptation term $\mathcal{L}_{dom}$ in the original manuscript's Section 2.7. We will ensure that the manuscript's Section 2.7 is updated to include this precise formulation and the necessary context regarding its optimization.

The adversarial training process employs a domain adversarial loss ($\mathcal{L}_{dom}$), formally defined as the binary cross-entropy (BCE) over the two domains:

$$\mathcal{L}_{dom} = \mathbb{E}_{x^s \sim \Phi^s}[\log D\,(H_s^{sh})] + \mathbb{E}_{x^t \sim \Phi^t}[\log\,(1 - D(H_t^{sh}))]$$

Where Φ^s and Φ^t denote the distributions of the bulk RNA-seq (source) and scRNA-seq (target) data, respectively, and $D(\cdot)$ is the probability output by the domain discriminator. This loss term governs the adversarial training through the following dual objectives, which form a minimax game:

Table R3-1

Component	Goal	Optimization Objective	Mechanism
Domain Discriminator	Domain Distinction	Maximize $\mathcal{L}_{dom}$	Domain discriminator is trained to correctly identify the domain source (y=1 for source, y=0 for target) based on the shared features.
Shared Encoder	Domain Confusion	Minimize $\mathcal{L}_{dom}$	Shared encoder is trained (via the Gradient Reversal Layer, GRL) to produce features that are indistinguishable to domain discriminator, effectively achieving feature invariance.

By optimizing this adversarial objective, the shared encoder is compelled to map the bulk and single-cell graph representations into a unified, domain-invariant subspace, which is essential for mitigating the graph-level domain shift and enabling robust knowledge transfer.

"The adversarial training process utilizes a domain adversarial loss ($\mathcal{L}_{dom}$), formally defined as:

$$\mathcal{L}_{dom} = \mathbb{E}_{x^s \sim \Phi^s}[\log D\,(H_s^{sh})] + \mathbb{E}_{x^t \sim \Phi^t}[\log\,(1 - D(H_t^{sh}))]$$

where Φ^s and Φ^t denote the distributions of the bulk RNA-seq and scRNA-seq data, respectively. Through this adversarial optimization, the domain discriminator aims to maximize $\mathcal{L}_{dom}$, enhancing its ability to differentiate domains, while the shared encoder attempts to minimize $\mathcal{L}_{dom}$, ensuring feature invariance." ***(Page 13, Line 453-458)***

Comment 3:

3. There are many models developed in the past two years, but the authors only selected scDEAL, SCAD, and some general machine learning algorithms as baseline methods. It is confusing, and the conclusion would be more comprehensive and reliable if the author compared the proposed model with a more recently developed method.

Response: We sincerely thank you for this insightful comment. We agree that benchmarking against the most recently developed single-cell drug response prediction models is essential to solidify the current state-of-the-art claim of DAGFormer.

In response, we have implemented and rigorously tested two highly relevant, recently published methods:

- scAdaDrug (Published August 2025, IEEE Journal of Biomedical and Health Informatics): Predicts drug sensitivity using Adversarial Multi-source Domain Adaptation.
- scGSDR (Published October 2025 in Nature Communications Biology): Performs pharmacological profiling by harnessing gene semantics.

The results of this expanded benchmark demonstrate that DAGFormer maintains its superior performance across all tested drug conditions, validating its core

architectural choices. More importantly, the comparison highlights the key methodological distinctions:

1. Distinction from scAdaDrug (Feature-based Alignment):

- scAdaDrug uses an Autoencoder and adaptive weighting mechanism to align feature vectors derived from multiple source domains. Its focus is on reducing the distance between feature embeddings from different datasets.

- DAGFormer's advantage is structural: we recognize that the domain shift is not only in the feature space but in the data structure itself (bulk data averages out topological information, while scRNA-seq reveals it). DAGFormer employs Graph Domain Adaptation (GDA), the first approach to our knowledge that adversarially aligns the topology of the bulk graph and the single-cell graph, ensuring that learned interactions are robust across domains.

2. Distinction from scGSDR (Knowledge-based Feature Engineering):

- scGSDR improves feature quality by integrating gene semantics and pathway knowledge, essentially creating a highly informed, high-quality feature vector representation.

- While effective, scGSDR still treats cells as isolated entities and does not model the crucial intercellular interactions inherent in the tumor microenvironment—a limitation DAGFormer explicitly overcomes. DAGFormer uses an graph tansformer to dynamically model these relationships, leading to a more comprehensive prediction model.

As demonstrated in Fig.4 and Fig.5 in the manuscript, DAGFormer consistently maintains its superior predictive performance. A clear difference in capability can be seen when examining the mean scores across all ten drug datasets for the deep learning models:

- The average ACC, AUC, and AUPR for scAdaDrug were 0.632, 0.667, and 0.697, respectively.

- The average ACC, AUC, and AUPR for scGSDR were 0.777, 0.840, and 0.875, respectively.

- In contrast, DAGFormer achieved significantly higher average scores of 0.902 (ACC), 0.935 (AUC), and 0.938 (AUPR).

While scGSDR proves to be a very strong competitor, ranking as the second-best deep learning method by a clear margin, DAGFormer still yields a substantial performance improvement across all key metrics. This result emphatically demonstrates the effectiveness of DAGFormer's novel architecture—specifically, its use of Graph Structure Modeling and Graph Domain Adaptation to address single-cell heterogeneity and topological domain shift—making it the superior choice even when benchmarked against the newest advanced methodologies.

“Recently, more specialized DL frameworks have emerged to tackle increasingly complex challenges in this domain, with a focus on either enhancing biological feature quality or refining domain adaptation techniques. For instance, models such as scAdaDrug\cite{scAdaDrug} emphasize advanced multi-source domain adaptation to improve transfer robustness, while scGSDR\cite{scGSDR} enhances predictive power by explicitly incorporating gene semantics and pathway knowledge to produce more informative feature representations. These recent developments illustrate a shift toward biologically informed and structurally adaptive deep learning strategies, setting the stage for further innovations such as DAGFormer.” ***(Page 3, Line 59-68)***

“scAdaDrug: A deep transfer learning model that predicts drug sensitivity using Adversarial Multi-source Domain Adaptation. It aligns feature vectors derived from multiple source domains, focusing on reducing the distributional distance between feature embeddings.

scGSDR: A recent pharmacological profiling model that enhances feature quality by harnessing gene semantics and pathway knowledge. It improves prediction by utilizing a highly informed, high-quality feature vector representation derived from biological prior knowledge.

XGBoost (Extreme Gradient Boosting): A highly optimized gradient boosting framework that is renowned for its speed and performance in classification tasks.

LightGBM (Light Gradient Boosting Machine): Another highly efficient and scalable gradient boosting framework that uses gradient-based one-side sampling and exclusive feature bundling to handle large datasets.” ***(Page 15, Line 512-542)***

“The results demonstrate that DAGFormer consistently achieves state-of-the-art performance across all seven drugs, outperforming both traditional machine learning models and the most recent deep learning baselines.The non-deep learning models, including XGBoost and LightGBM, show poor predictive capability, confirming their inability to capture the high-dimensional complexity of pre-treatment transcriptional profiles.” ***(Page 22, Line 721-726)***

“Among the deep learning baselines, the performance gap between DAGFormer and its strongest competitor, scGSDR, is decisive. For example, for Sorafenib, DAGFormer achieves AUC = 0.987 and AUPR = 0.988, compared to scGSDR's AUC = 0.771 and AUPR = 0.794. Similarly, for NVP-TAE684, DAGFormer

improves the AUC by 26.1% and the AUPR by 20.9% over the next-best model (scAdaDrug). Crucially, even against the strong classical domain adaptation competitor SCAD, DAGFormer shows consistent superiority. For Gefitinib, where the SCAD_WEIGHT variant achieves an impressive secondary performance of AUC = 0.967 and AUPR = 0.973, DAGFormer surpasses this result, reaching AUC = 0.989 and AUPR = 0.989. This consistently superior performance is directly attributable to the core innovations of DAGFormer, which integrates the modeling of cell–cell interactions to capture complex topological structures with a Dual-Domain Collaborative Decoupling–Fusion strategy. The latter employs private encoders to preserve unique domain-specific biological signals, while the Graph Domain Adaptation module aligns the shared, invariant features. Together, these mechanisms form a comprehensive structural and feature-level framework that makes DAGFormer the most robust model for predicting drug resistance." ***(Page 22, Line 727-742)***

Method	Etoposide			PLX4720_A375			PLX4720_451Lu			Mean Score		
	ACC	AUC	AUPR	ACC	AUC	AUPR	ACC	AUC	AUPR	ACC	AUC	AUPR
AdaBoost	0.548	0.501	0.562	0.472	0.430	0.570	0.523	0.497	0.499	0.514	0.476	0.543
XGBOOST	0.452	0.482	0.540	0.426	0.837	0.845	0.516	0.416	0.441	0.465	0.578	0.609
LightGBM	0.452	0.593	0.623	0.426	0.848	0.864	0.516	0.392	0.399	0.465	0.611	0.629
Random Forest	0.424	0.441	0.505	0.435	0.861	0.888	0.523	0.435	0.433	0.461	0.579	0.609
Logistic Regression	0.453	0.546	0.580	0.556	0.769	0.814	0.497	0.418	0.432	0.502	0.578	0.609
Decision Tree	0.524	0.496	0.547	0.639	0.632	0.652	0.490	0.485	0.470	0.551	0.538	0.556
Naive Bayes	0.444	0.550	0.549	0.611	0.724	0.789	0.374	0.335	0.403	0.476	0.536	0.580
SCAD_SMOTE	-	0.694	0.736	-	0.825	0.875	-	0.381	0.461	-	0.633	0.691
SCAD_WEIGHT	-	0.669	0.694	-	0.694	0.769	-	0.381	0.478	-	0.581	0.647
scGSDR	0.595	0.652	0.633	0.857	0.964	0.957	0.581	0.542	0.617	0.677	0.719	0.736
scDEAL	0.452	0.476	0.407	0.426	0.617	0.427	0.523	0.484	0.546	0.467	0.526	0.460
scAdaDrug	0.607	0.629	0.669	0.727	0.812	0.894	0.562	0.559	0.529	0.632	0.667	0.697
DAGFormer	0.835	0.843	0.908	0.972	1.000	1.000	0.587	0.629	0.600	0.798	0.824	0.836

Fig 4.Performance comparison of different methods across drugs (post-treatment).

	Gefitinib			Afatinib			AR-42			Cetuximab			NVP-TAE684			Sorafenib			Vorinostat		
Method	ACC	AUC	AUPR	ACC	AUC	AUPR	ACC	AUC	AUPR	ACC	AUC	AUPR	ACC	AUC	AUPR	ACC	AUC	AUPR	ACC	AUC	AUPR
AdaBoost	0.485	0.37	0.435	0.467	0.492	0.547	0.470	0.264	0.373	0.525	0.572	0.603	0.475	0.489	0.496	0.475	0.489	0.522	0.576	0.676	0.676
XGBOOST	0.500	0.761	0.774	0.500	0.729	0.731	0.500	0.372	0.475	0.500	0.590	0.619	0.500	0.564	0.534	0.500	0.526	0.554	0.500	0.713	0.696
LightGBM	0.500	0.736	0.723	0.500	0.602	0.597	0.500	0.386	0.444	0.500	0.612	0.609	0.500	0.546	0.539	0.500	0.782	0.786	0.500	0.782	0.786
SVM	0.530	0.466	0.526	0.533	0.512	0.581	0.485	0.600	0.602	0.467	0.460	0.501	0.500	0.458	0.502	0.525	0.594	0.600	0.500	0.615	0.632
Naive Bayes	0.515	0.553	0.527	0.517	0.535	0.515	0.530	0.680	0.668	0.500	0.498	0.495	0.408	0.408	0.452	0.608	0.664	0.629	0.515	0.673	0.613
Random Forest	0.606	0.694	0.742	0.642	0.770	0.774	0.500	0.762	0.718	0.541	0.664	0.668	0.550	0.617	0.613	0.500	0.589	0.581	0.500	0.768	0.757
Logistic Regression	0.636	0.772	0.728	0.558	0.585	0.577	0.485	0.652	0.638	0.475	0.430	0.465	0.558	0.680	0.677	0.550	0.735	0.764	0.500	0.591	0.573
Decision Tree	0.667	0.667	0.608	0.525	0.525	0.513	0.485	0.485	0.493	0.517	0.517	0.509	0.375	0.375	0.457	0.433	0.433	0.469	0.636	0.636	0.624
scDEAL	0.500	0.683	0.708	0.467	0.353	0.157	0.500	0.531	0.370	0.500	0.468	0.447	0.500	0.698	0.744	0.500	0.568	0.558	0.500	0.486	0.283
scAdadrug	0.800	0.840	0.878	0.583	0.725	0.720	0.500	0.550	0.541	0.534	0.798	0.784	0.437	0.734	0.821	0.388	0.339	0.420	0.550	0.590	0.638
SCAD_SMOTE	-	0.773	0.824	-	0.925	0.938	-	0.801	0.826	-	0.798	0.843	-	0.654	0.695	-	0.771	0.794	-	0.942	0.958
SCAD_WEIGHT	-	0.967	0.973	-	0.880	0.889	-	0.968	0.970	-	0.923	0.921	-	0.598	0.664	-	0.582	0.627	-	0.902	0.904
scGSDR	0.769	0.952	0.948	0.833	0.923	0.920	0.786	0.896	0.938	0.833	0.871	0.833	0.792	0.821	0.868	0.833	0.832	0.890	0.769	0.929	0.959
DAGFormer	0.955	0.989	0.989	0.967	0.989	0.992	0.970	0.998	0.998	0.892	0.957	0.958	0.950	0.959	0.953	0.925	0.987	0.988	0.970	0.996	0.997

Fig 5.Performance comparison of different methods across drugs (post-treatment).

**Comment 4:**
4. The introduction states DAGFormer is "the first DL model" to explore multiple cellular-graph topologies for single-cell drug response with bulk integration; unless a comprehensive survey confirms this, the phrasing should be softened.

Response: We appreciate the reviewer's attention to the precision of our language. To maintain scientific rigor and focus on the unique nature of our methodology, we will revise the phrasing to reflect that DAGFormer represents a rare approach in this specific domain.

We will revise the statement in the introduction to reflect this more objective claim:"To the best of our knowledge, DAGFormer is a rare deep learning model that systematically explores and assesses different methods for constructing cellular-cellular interaction network topologies in predicting cancer drug response at the single-cell level through integration with bulk RNA-seq data."***(Page 3, Line 86-89)***

This revised wording asserts the novelty of our systematic, graph-based investigation without relying on an absolute claim of temporal precedence. We will apply this softened phrasing throughout the manuscript where applicable.

**Comment 5:**
5. The graph construction processing is time-consuming; the author should discuss the proposed models' time complexity and storage burden.

Response: Thank you for raising the crucial issues of time complexity and storage burden. We agree that discussing these aspects is vital, especially since our approach involves KNN graph construction, which is known to be computationally intensive. Our model's computational cost is dominated by two primary stages: Graph Construction (Preprocessing) and Model Training (Deep Learning).

The computational cost of graph construction is primarily dominated by the initial, static construction of the cellular neighbor graphs, G_s (for Bulk data) and G_t

(for scRNA-seq data). This process is executed only once prior to model training. The complexity is governed by the two largest data scales: the number of samples (N) and the number of shared genes (g , where g $\approx$ 10,000).

Table R3-2

Component	Samples (N)	Time Complexity	Note
Cellular KNN Graph Construction	N (cells/cell lines)	$O(N^2 g + N^2 \log N)$	Dominated by distance calculation ($N^2 g$) across all g genes and sorting to find K neighbors.

For our datasets, the number of samples N is moderate (e.g., Bulk $N_b \approx 850$ cell lines; scRNA-seq $N_s \approx 120$ single cells). Since this high-complexity step is pre-computed, the total time cost remains manageable, typically taking minutes depending on g $\approx$ 10,000.

The storage burden is critical because our implementation uses full-batch training. This requires the entire data and graph structure to fit into the GPU's high VRAM. The storage complexity demonstrates that this strategy is feasible in Table R3-3.

"*The cellular neighbor graph construction is the most computationally demanding step, dominated by the $O(N^2 \cdot g)$ time complexity required for distance calculation and KNN identification, where N is the number of samples (cells/cell lines) and g is the number of shared genes. Since this high-complexity process is pre-computed once prior to model training, the total time cost remains manageable (typically taking minutes for our moderate N). The storage burden is also critical due to our full-batch training strategy. While the gene expression features require $O(N \cdot g)$ storage, the graph adjacency matrix is stored in a highly efficient sparse format, resulting in a minimal storage complexity of $O(N \cdot K)$ (where K is the number of neighbors).*"***(Page 17, Line 599-607)***

Table R3-3

Component	Storage Complexity	Note
Gene Expression Features (X)	$O(N \cdot g)$	For the Bulk dataset, $N \approx 850$ cells and $g \approx 10{,}000$ genes. This is large but feasible for high VRAM GPUs.
Graph Adjacency Matrix (G)	$O(N \cdot K)$	Stored in sparse format (where K=15). This is highly efficient and minimizes the storage overhead compared to a dense N^2 matrix.

Comment 6:

6. The author needs to provide more details about the datasets in the article.

Response: Thank you for your suggestion. We have expanded the description of our source and target domains, meticulously clarifying how the single-cell drug response labels were derived for our two distinct experimental scenarios.

1. Source Domain: Bulk RNA-seq Data

The source domain data is sourced from the GDSC (Genomics of Drug Sensitivity in Cancer) database. This broad, readily accessible dataset is used for model

training to learn general drug response knowledge.

The data undergoes preprocessing starting with RMA normalization7. Crucially, the continuous IC50 values (half-maximal inhibitory concentration) are transformed into binary labels ('sensitive' or 'resistant') using the LOBIco (logistic optimization binary classification) algorithm.

2. Target Domain: Single-Cell RNA-seq Data

The target domain consists of multiple scRNA-seq datasets used for predicting cancer drug responses at the single-cell level. This domain provides higher-resolution information, but its drug response data is often more scarce. Knowledge learned from the source domain is transferred here to make single-cell predictions. Data was collected from three primary sources:

- CCLE Dataset: Includes 10x Genomics sequencing data for the JHU006 and SCC47 cell lines, used to assess sensitivity to Gefitinib, Afatinib, AR-42, Cetuximab, NVP-TAE684, Sorafenib, and Vorinostat.
- GSE149215 Dataset: Provides data for the PC9 cell line, which includes untreated sample cells, specifically used for Etoposide response analysis.
- GSE108383 Dataset: Employs the SMART-seq platform for the A375 and 451Lu cell lines, focusing specifically on assessing sensitivity to PLX4720.

The scRNA-seq data underwent rigorous quality control (QC) (e.g., filtering out cells with high mitochondrial gene expression), followed by normalization, logarithmic transformation, and Z-score standardization. To ensure compatibility, only genes present in both the Bulk and scRNA-seq datasets were retained for training. As shown in Table 2 of the article, these datasets span a wide array of drugs and platforms, providing comprehensive support for single-cell drug sensitivity analysis.

Besides, The label derivation method is explicitly determined by the experimental context in Table R3-4.

We have updated the manuscript (Sections 2.1) to explicitly state these experimental mappings and label derivation rules for each scenario, thereby providing the full context required for interpreting the results in Fig.4 and Fig.5 of the article.

Table R3-4

Experimental Scenario	Label Derivation Methodology	Corresponding Results Table
Post-treatment (Acquired Resistance)	This scenario evaluates cells after drug exposure. Parental cells (sequenced from drug-untreated lines) are classified as sensitive cells, while drug-tolerant cells that survived drug exposure are categorized as resistant cells.	Fig.4 (Etoposide, PLX4720_A375, PLX4720_451Lu)
Pre-treatment (Inherent Resistance)	This scenario evaluates cells prior to drug exposure, assuming the existence of pre-existing drug-resistant subpopulations. Cells are labeled based on inferred or established drug-resistance markers within the untreated population.	Fig.5 (Gefitinib, Cetuximab, Afatinib, AR-42, NVP-TAE684, Sorafenib, Vorinostat)

"The single-cell RNA-seq data within the target domain is utilized to evaluate two critical experimental scenarios, which reflect the two primary mechanisms of tumor drug resistance: Acquired Resistance and Inherent Resistance. The first is the Post-treatment (Acquired Resistance) scenario. This approach focuses on evaluating the state of cells after drug exposure, defining sensitivity and resistance by comparing drug-untreated parental cells with drug-tolerant cells that survived the drug treatment. The results for this type of analysis are presented in Fig. 4, covering drug responses for agents such as Etoposide and PLX4720. The second is the Pre-treatment (Inherent Resistance) scenario. This approach assesses the state of cells prior to drug exposure, primarily focusing on the analysis and prediction of pre-existing resistant subpopulations within the untreated population. The research results for this scenario are shown in Fig. 5, encompassing responses to various drugs including Gefitinib and Cetuximab. Through the classification into these two distinct experimental scenarios, we are able to gain a deeper, single-cell level understanding of the mechanisms underlying different types of drug resistance." ***(Page 6, Line 163-177)***

**Comment 7:**
7. The GitHub page needs to be improved, and a tutorial is also necessary.

Response: Thank you for your valuable feedback regarding the usability of our GitHub repository. We agree that a well-structured repository and a comprehensive tutorial are essential for maximizing the impact and reproducibility of DAGFormer. We have taken immediate action to significantly improve the GitHub page and user documentation:

We enhanced the github structure and content:

- Explicit Reproducibility Details: We have added a dedicated section detailing the exact Computational Environment used for the reported results, including the specific versions of Python, PyTorch, DGL, and CUDA dependencies, as requested by previous reviewers .
- Parameter Settings: The default hyperparameters used for all benchmarking runs are now clearly listed.
- Model Architecture Overview: A clear, concise overview of the DAGFormer framework and its innovative dual-encoder structure is provided.
- Data Availability Statement: Clear links and instructions for accessing the preprocessed GDSC (source) and scRNA-seq (target) datasets are included.

We believe these improvements significantly lower the entry barrier for users and enhance the reproducibility of our reported findings. The updated repository is available at https://github.com/yanfen-git/DAGFormer.

**Comment 8:**
8. There are some errors in equation (20), especially the character "where" should not be indented two spaces to the right at the beginning of the paragraph.

Response: Thank you for your careful review. We apologize for the typographical error in Equation (20)'s description. We confirm that the word "where" will be corrected by removing the two-space indentation at the beginning of the paragraph following Equation (20) in the revised manuscript. This ensures proper

formatting and adherence to standard typesetting conventions.

**Comment 9:**
9. The author needs to improve figure clarity (fonts, resolution, consistent labels).

Response: Thank you for your valuable feedback regarding the clarity and presentation of our figures. We fully agree that high-quality visuals are essential for effective scientific communication.

We have addressed figure presentation comprehensively across the manuscript:

1. Increased Font Size and Readability: We have globally increased the font size for titles, axis labels, and legends in all figures, particularly in the UMAP visualizations (Figure 4), to ensure they are easily readable, as previously suggested .

2. Improved Consistency and Labeling: We have ensured consistent and accurate labeling, specifically correcting the title of the central UMAP visualization in Figure 4 to PLX4720_A375 to avoid ambiguity.

3. Enhanced Visual Palette: We have implemented a unified and aesthetically optimized color palette across all comparison figures (e.g., Figure 5) to improve contrast, ensure consistency, and enhance the clarity of the results, especially in ablation studies.

These improvements significantly enhance the aesthetic quality, resolution, and overall interpretability of all figures in the revised manuscript.
