# Supplementary material for: DAGFormer: A graph-based domain adaptation approach for single-cell cancer drug response prediction
Source: PLoS Comput Biol. 2025 Dec 19;21(12):e1013832. doi: 10.1371/journal.pcbi.1013832 (PMC12795466; doi:10.1371/journal.pcbi.1013832)
Supplement: Attachment — Submitted filename: Review of Manuscript DAGFormer.docx [file pcbi.1013832.s001.docx]

**Review of Manuscript: DAGFormer, a novel deep learning framework for predicting single-cell drug responses by integrating bulk and single-cell RNA-seq data**

**Summary**

This manuscript by Yan et al. presents DAGFormer, a novel deep learning framework for predicting single-cell drug responses by integrating bulk and single-cell RNA-seq data. The authors address key limitations of 2 previous methods by 1) incorporating cellular relationship graphs as constraints when encoding features of each cell/cell-line and 2) employing a dual-domain architecture to preserve both domain-shared and domain-private features, specifically accomplished via a combination of an adversarial training scheme on shared features and enforcing orthogonality between shared and private features.

Through comprehensive experiments on 9 different drugs, the authors demonstrate that DAGFormer significantly outperforms a suite of classical and state-of-the-art baseline models. The authors further present ablation studies to provide adequate support and reasoning for their model setup and choice. This work presents an improved approach to transferring characteristics learnt from bulk RNA-seq data to scRNA-seq. The results are compelling, though are several points of concern as listed below:

**Major Points**

1. **Unclear training and testing scheme.** it is unclear how the training data and test data for each drug was set up. From the results and data descriptions, it seems that the scRNA-seq data was only used for testing and evaluations, however the model explicitly calculates losses for scRNA-seq derived features such as the graph reconstruction loss and adversarial loss between bulk and single cell embeddings. The authors should provide a detailed description of how training and testing data was set up for a drug/experiment (assuming it is the same for the other drugs).
2. As a follow-up to the previous point, if all single cells were utilized for training (similar to scDEAL), it would be interesting to see the results of a portion of single cells that are left out of training entirely (no contribution to model losses) and only used for evaluation (especially for drugs like Etoposide that have more single cells).
3. It is also unclear whether the authors trained with the mini-batches or the entire data all at once. If mini-batches were used, it would be interesting to see the effect of batch-size on results since smaller batch-sizes may not contain enough cellular relationships to construct meaningful embeddings.
4. **The process of graph construction is still unclear.** For instance, is it correct to assume that graph construction for the bulk data was performed with bulk RNA-seq of all cell lines prior to any training and not on-the-fly within each batch, the descriptions are vague.
5. **Unclear description of oversampling and under-sampling (line 127 -line 133)**, the authors mention both oversampling minority classes to 50% of majority class and under-sampling majority class to 50% of minority class, which does not make sense. If both sampling techniques were used, the authors could include supplementary figures to show the differences in results.
6. The authors cite Ho et al. to explain the performance disparity for PLX4720 between the A375 and 451Lu cell lines. However, the current explanation is indirect. The authors should more explicitly state how the cited work explains the *difference in transcriptional separability* between sensitive and resistant populations in these two specific cell lines, as this is the likely factor driving the model's differential performance.
7. **Treatment of Drug Response as a Binary Problem:** The study, like many others in the field, simplifies the drug response prediction task into a binary classification ('sensitive' vs. 'resistant') by transforming continuous IC50 values. While the authors acknowledge this as a limitation in their conclusion, this simplification can mask important nuances in drug sensitivity. It would strengthen the paper to include a brief discussion on the sensitivity of the results to the binarization method (LOBIco) or to elaborate on why a regression-based approach was not pursued. Could the framework be adapted to predict continuous IC50 values directly?

**Minor Points**

1. **Justification of K in KNN:** The authors state that K=15 was selected for the KNN graph construction based on experimental results that yielded the best performance. It would improve transparency to include a supplementary figure or table showing the model's performance across the tested range of K values (5, 10, 15, 30).
2. **Figure Readability:** In Figure 4, the UMAP titles and axis fonts are rather small, could be made bigger.
3. Replace Adaboost with a better boosting method such as XGBoost or LightGBM for comparison.
4. **Clarity on Baseline Results:** The manuscript notes that results for the SCAD model were directly adopted from the original publication. While this is a common practice, it is worth noting for the record, as minor differences in data preprocessing could influence performance.
5. Table 3, second row PLX4720 should be PLX4720_A375, as is also the case for Figure 4 center column of UMAPs.
6. The manuscript is well-written, but a few minor typographical errors were noted.
   - GSE14921 in **line 460 and 481** should be GSE149215
   - Line 512 – 514, perhaps the authors should add “and used the scRNA-seq data”
