# Supplementary material for: DAGFormer: A graph-based domain adaptation approach for single-cell cancer drug response prediction
Source: PLoS Comput Biol. 2025 Dec 19;21(12):e1013832. doi: 10.1371/journal.pcbi.1013832 (PMC12795466; doi:10.1371/journal.pcbi.1013832)
Supplement: Attachment — Submitted filename: PCOMPBIOL-D-25-01609-R1-response.docx [file pcbi.1013832.s003.docx]

**Response to Revision 1 of DAGFormer: A graph-based domain adaptation approach for single-cell cancer drug response prediction**

**Recommendation:** Accept

**Comments to the Authors:** After reviewing the revised manuscript and the authors’ detailed point-by-point response, it is clear that the authors have done a commendable job addressing all concerns raised in the previous round of review. The revision has significantly improved the clarity, robustness, and methodological rigor of the study.

This was most notably highlighted in the following aspects:

- The inclusion of the new ablation experiment on the Etoposide dataset (Section 3.8) provides critical evidence regarding the model's generalization capability on strictly unseen data. This directly addresses my concern regarding the training/testing scheme.
- The authors not only replaced AdaBoost with state-of-the-art tree-based models (XGBoost and LightGBM) but also included recent deep learning baselines (scAdaDrug and scGSDR). These benchmark additions firmly establish DAGFormer's performance relative to the current field.
- The added "Problem Definition" section and the detailed explanation of the static graph construction have resolved the ambiguity regarding the experimental setup. The clarification on the sampling procedure was also appreciated.
- The updated figures, particularly the improved readability and labeling in Figure 4 (now corrected to PLX4720_A375), are much clearer.

I have no further comments or requests. I recommend the manuscript for publication in its current form.
